# Transcription factor activity rhythms and tissue-specific chromatin interactions explain circadian gene expression across organs

**DOI:** 10.1101/gr.222430.117

**Published:** 2018-02

**Authors:** Jake Yeung, Jérôme Mermet, Céline Jouffe, Julien Marquis, Aline Charpagne, Frédéric Gachon, Felix Naef

**Affiliations:** 1 Institute of Bioengineering, School of Life Sciences, Ecole Polytechnique Fédérale de Lausanne (EPFL), Lausanne, CH-1015, Switzerland;; 2 Department of Diabetes and Circadian Rhythms, Nestlé Institute of Health Sciences, CH-1015 Lausanne, Switzerland;; 3 Functional Genomics, Nestlé Institute of Health Sciences, CH-1015 Lausanne, Switzerland;; 4 Faculty of Life Sciences, Ecole Polytechnique Fédérale de Lausanne (EPFL), CH-1015 Lausanne, Switzerland

## Abstract

Temporal control of physiology requires the interplay between gene networks involved in daily timekeeping and tissue function across different organs. How the circadian clock interweaves with tissue-specific transcriptional programs is poorly understood. Here, we dissected temporal and tissue-specific regulation at multiple gene regulatory layers by examining mouse tissues with an intact or disrupted clock over time. Integrated analysis uncovered two distinct regulatory modes underlying tissue-specific rhythms: tissue-specific oscillations in transcription factor (TF) activity, which were linked to feeding-fasting cycles in liver and sodium homeostasis in kidney; and colocalized binding of clock and tissue-specific transcription factors at distal enhancers. Chromosome conformation capture (4C-seq) in liver and kidney identified liver-specific chromatin loops that recruited clock-bound enhancers to promoters to regulate liver-specific transcriptional rhythms. Furthermore, this looping was remarkably promoter-specific on the scale of less than 10 kilobases (kb). Enhancers can contact a rhythmic promoter while looping out nearby nonrhythmic alternative promoters, confining rhythmic enhancer activity to specific promoters. These findings suggest that chromatin folding enables the clock to regulate rhythmic transcription of specific promoters to output temporal transcriptional programs tailored to different tissues.

A mammalian internal timing system, known as the circadian clock, orchestrates temporal physiology in organs to anticipate daily environmental cycles (Dibner and Schibler 2015). Individual cells within organs contain a molecular oscillator that, together with rhythmic systemic signals such as hormones, temperature, and feeding behavior, collectively drive diurnal oscillations in gene expression and physiology (Lamia et al. 2008; Reinke et al. 2008; Cho et al. 2012; Vollmers et al. 2012). Remarkably, the circadian clock impinges on many gene regulatory layers, from transcriptional and post-transcriptional processes, translation efficiency, to translational and post-translational processes (Mermet et al. 2016).

Transcriptome analysis of different cell types and tissues has highlighted the breadth of tissue-specific transcriptional regulation (Merkin et al. 2012; Yue et al. 2014). However, physiological processes are dynamic at the timescale of hours and often under circadian control, such as hormone secretion, drug and xenobiotic metabolism, and glucose homeostasis (Takahashi et al. 2008). Adding the temporal dimension to tissue-specific gene regulation is needed for an integrated understanding of physiology.

Chronobiology studies have shown that tissues utilize the circadian clock to drive tissue-specific rhythmic gene expression (Storch et al. 2002; Korenčič et al. 2014; Zhang et al. 2014), presumably to schedule physiological functions to optimal times of day. Indeed, genetic ablation of the circadian clock in different tissues can lead to divergent pathologies, such as diabetes in pancreas-specific *Bmal1* knockout (KO) and fasting hypoglycemia in liver-specific *Bmal1* KO, suggesting that the clock interweaves with tissue-specific transcriptional programs (Bass and Lazar 2016), but how diurnal and tissue-dependent regulatory landscapes interact to generate tissue-specific rhythms is poorly understood.

## Results

### Contributions of tissue, daily time, and circadian clock to global variance in mRNA expression

To estimate the respective contributions of tissues, daily time, and circadian clock to global variance in gene expression, we analyzed available temporal transcriptomes across 11 tissues in WT mice (Zhang et al. 2014) and generated temporal RNA-seq data of liver and kidney from *Bmal1* KO mice and WT littermates (Supplemental Tables S1, S2; Methods). The Zhang et al. data set was obtained under dark–dark (DD), ad libitum feeding, sampled every 2 h. The liver and kidney *Bmal1* KO and WT data sets were obtained under light–dark (LD), night-restricted feeding conditions, sampled every 4 h.

**Figure 1.**
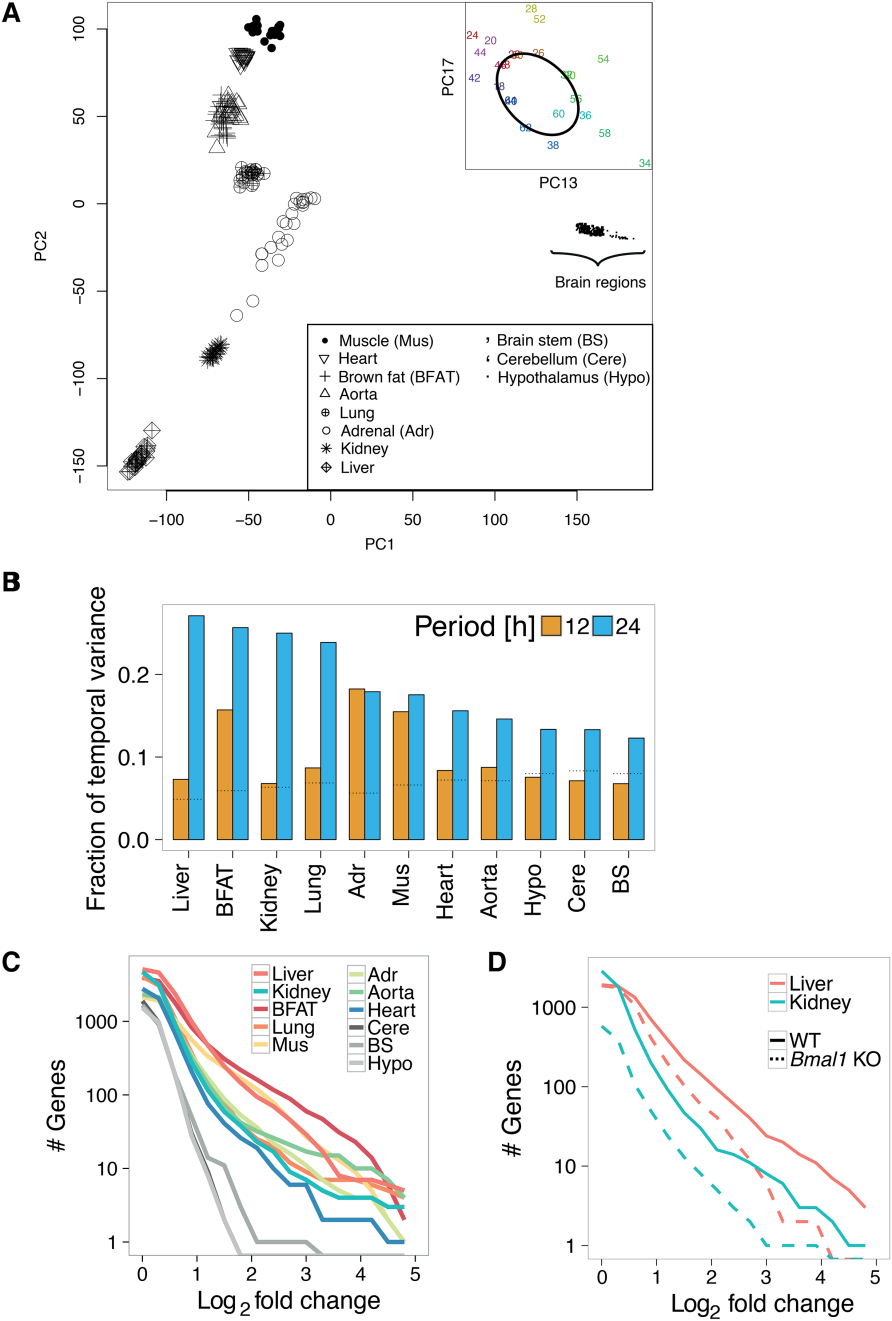
Contribution of tissue, daily time, and circadian clock to global variance in mRNA expression. (*A*) Principal component analysis (PCA) across 11 WT tissues sampled over 2 d. PC1 and PC2 show clustering of samples by tissues; each point represents a tissue sample (see key) at a specific time point (not labeled). (*Inset*) Loadings for PC13 and PC17 for the liver samples labeled with circadian time (CT), showing temporal variation along an elliptic path. Labels indicate CT time; samples that are 24 h apart are in the same color. (*B*) Fractions of temporal variance in each tissue explained by 24- and 12-h periods, obtained by applying spectral analysis genome-wide for each tissue. Dotted horizontal lines represent the expected background level, assuming white noise. (*C*,*D*) Cumulative number of rhythmic genes (*P* < 0.01, harmonic regression) with log_2_ fold change larger than the value on the *x*-axis. (*C*) Analysis on 11 WT tissues. (*D*) Analysis on four conditions: *Bmal1* KO mice and WT littermates in liver and kidney.

To avoid mixing different experimental designs (e.g., temporal resolution and number of repeats) (Deckard et al. 2013; Li et al. 2015), we analyzed these data sets separately. We performed principal component analysis (PCA) on the entire set of conditions (11 tissues × 24 time points) to obtain an unbiased overview into the contributions of tissue- and time-specific variance. Most of the variance concerned differences in expression between tissues (Fig. 1A; Supplemental Fig. S1A–D). Temporal variance, in particular, 24-h periodicity, was present among a group of principal components carrying lower amounts of variance (Fig. 1A; Supplemental Fig. S1E–G). Focusing on genome-wide temporal variation within each tissue, we found that 24-h rhythms constituted the largest contribution of temporal variance, followed by 12-h rhythms, which were close to background levels for many tissues (Fig. 1B; Hughes et al. 2009). We thus focused the rest of our analysis on 24-h rhythms.

We analyzed the peak-to-trough amplitudes (hereafter also referred to as fold change) of 24-h rhythmic transcripts. Metabolic tissues, notably liver, brown fat, and skeletal muscle exhibited more (on the order of 100 transcripts) intermediate- to high-amplitude (between two- and 10-fold) transcript rhythms. Brain tissues showed virtually no rhythmic transcripts above fourfold (Fig. 1C). In liver and kidney of *Bmal1* KO mice, the number of rhythmic mRNAs was reduced by threefold compared to WT littermates. This effect increased for larger amplitudes. Few transcripts in tissues of *Bmal1* KO oscillated by more than 10-fold (Fig. 1D). Thus, a functional circadian clock is required for high-amplitude transcript rhythms across diverse tissues, while systemic signals regulate lower amplitude rhythms that persist in clock-deficient liver (Hughes et al. 2012; Atger et al. 2015; Sobel et al. 2017) and kidney (Nikolaeva et al. 2012).

### Combinatorics of rhythmic transcript expression across tissues and genotypes

**Figure 2.**
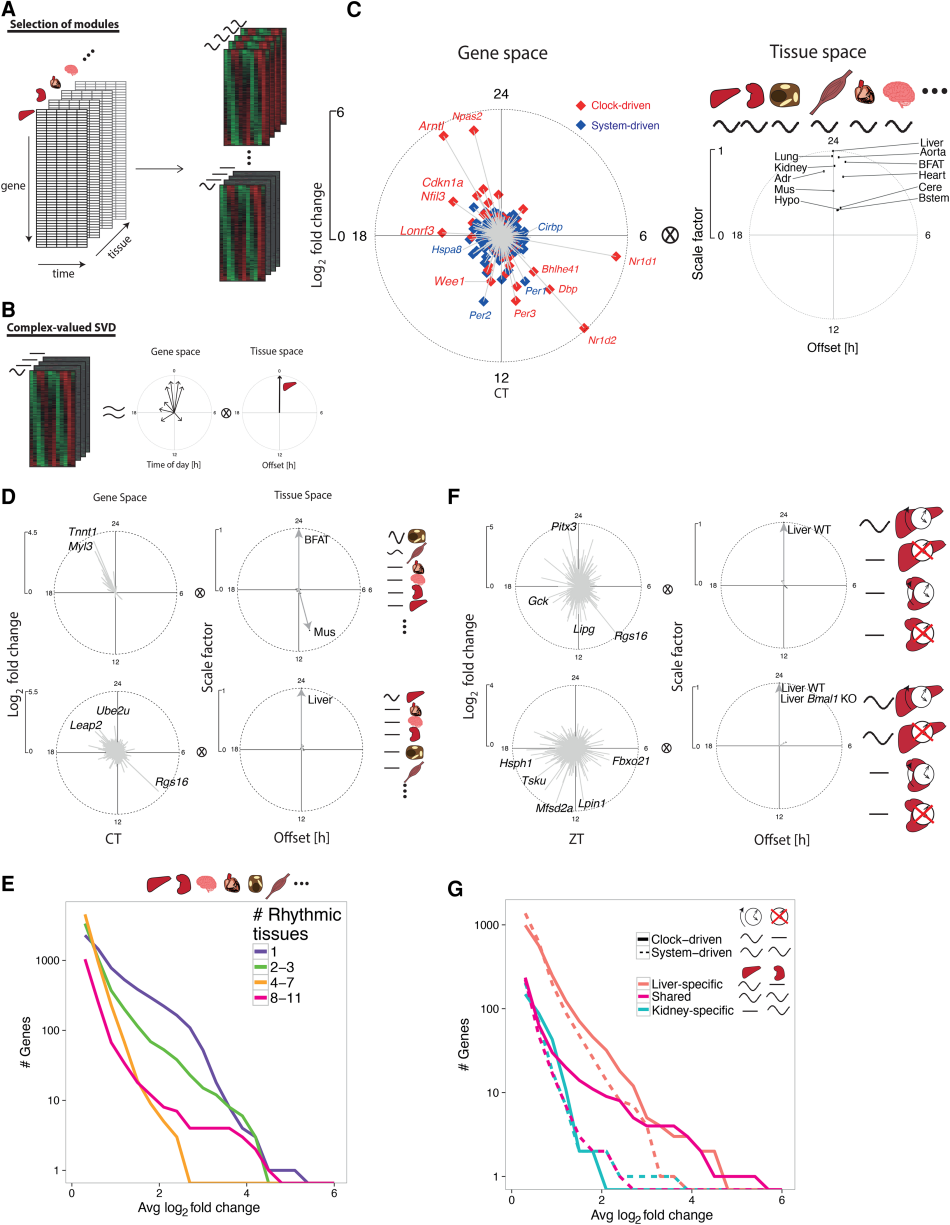
Combinatorics of rhythmic transcript expression across tissues and genotypes. (*A*) Schema for the model selection (MS) algorithm to identify rhythmic gene expression modules across tissues. Temporal transcriptomes of different tissues represented as a three-dimensional array (*left*). Gene modules are probabilistically assigned among different combinations of 24-h rhythms across tissues (e.g., tissue-specific or tissue-wide rhythms schematically shown on *right*). (*B*) Gene modules are summarized by the first component of complex-valued singular value decomposition (SVD) to highlight phase (peak time shown as the clockwise angle) and amplitude (log_2_ fold change shown as the radial distance) relationships between genes (gene space) and between tissues (tissue space). SVD representation is scaled such that the genes show log_2_ fold changes, while tissue vectors are scaled such that the highest amplitude tissue has length of 1 and a phase offset of 0 h. (*C*–*E*) MS applied to 11 WT tissues. (*C*) SVD representation of tissue-wide mRNA rhythms from the 11 tissues. Genes are labeled as system-driven (blue) or clock-driven (red) according to the comparison of the corresponding temporal profiles in *Bmal1* KO and WT littermates. (*D*) Examples of anti-phasic rhythms (brown fat and muscle, *n* = 20, first SVD component explains 81% of variance), and tissue-specific rhythms (liver, *n* = 846, first SVD component explains 59% of variance). Representative genes with large amplitudes are labeled. (*E*) Number of transcripts showing rhythms (*P*-value < 0.01, harmonic regression) in different numbers of tissues, in function of increasing peak to trough amplitudes on the *x*-axis. *x*-axis: average log_2_ fold change calculated from the identified rhythmic tissues. (*F*,*G*) MS applied to *Bmal1* KO and WT littermates in liver and kidney. (*F*) SVD representation of clock- (*top*, *n* = 991, 83% of variance) and system-driven (*bottom*, *n* = 1395, 84% of variance) liver-specific rhythms. (*G*) Number of transcripts showing clock- (solid) or system-driven (dotted) rhythms (*P*-value < 0.01, harmonic regression) in liver (red), kidney (blue), or both (magenta).

We reasoned that identifying sets of genes with shared rhythms across subsets of tissues would allow finding underlying regulatory mechanisms. We developed a model selection (MS) algorithm extending harmonic regression (Fisher 1929) to classify genes into modules sharing rhythmic mRNA profiles across subsets of tissues (Fig. 2A; Methods). Phase (time of peak) and amplitude (log_2_ fold change) relationships between genes and tissues are summarized using complex-valued singular value decomposition (SVD) (Fig. 2B; Methods). We applied MS to the 11 tissues, which identified gene modules involving rhythmic mRNA accumulation in nearly all tissues (tissue-wide) (Fig. 2C), in single tissues (tissue-specific), or in several tissues (tissue-restricted) (examples shown in Fig. 2D; Supplemental Fig. S2A; Supplemental Table S3).

The tissue-wide module contained a set of both clock- and system-driven rhythmic mRNAs, as determined by comparing *Bmal1* KO data in liver and kidney (Fig. 2C, left). Moreover, these transcripts oscillated in synchrony across all tissues and peaked at fixed times of day, although their amplitudes varied between tissues, with brain regions showing the smallest amplitudes (Fig. 2C, right). The clock drove synchronized oscillations at high amplitudes, notably clock genes (e.g., *Arntl, Npas2, Nr1d1,2*; note that *Arntl* and *Nr1d1,2* are also named *Bmal1* and *Rev-erba,b,* respectively), clock output genes (e.g., *Dbp, Nfil3*), and cell cycle regulators (*Cdkn1a* and *Wee1*) (Matsuo et al. 2003; Gréchez-Cassiau et al. 2008). Interestingly, clock genes *Per1,2* continued to oscillate in *Bmal1* KO in multiple tissues, extending previous studies in liver (Kornmann et al. 2007). Other clock-independent oscillations included mRNAs of heat- and cold-induced genes, such as *Hspa8* and *Cirbp* (Morf et al. 2012; Gotic et al. 2016)*,* that peaked 12 h apart near CT18 and CT6 (CT: circadian time; CT0 corresponds to subjective dawn and start of the resting phase; CT12 corresponds to subjective dusk and start of the activity phase), concomitantly with highs and lows in body temperature rhythms (Refinetti and Menaker 1992).

Tissue-restricted modules contained rhythmic transcripts that peaked in synchrony, such as in liver and kidney, or with fixed offsets, such as the nearly 12-h shifted rhythms in brown fat and skeletal muscle (Supplemental Fig. S3A). Overall, transcripts with large amplitudes (FC > 8) oscillated in either a few tissues (three or less) or tissue-wide (eight or more) (Fig. 2E).

To distinguish clock- and system-driven mRNA rhythms, we applied the MS algorithm to the liver and kidney transcriptomes in WT and *Bmal1* KO mice (Fig. 2F; Supplemental Fig. S3B; Supplemental Table S4). This separation identified clock- and system-driven modules that oscillated in liver but were flat in kidney (Fig. 2F), as exemplified by mRNAs of *Lipg* and *Lpin1* (Supplemental Fig. S2B). Indeed, both transcripts oscillated in WT liver with robust amplitudes, peaking near ZT11, but were flat in kidney (ZT: Zeitgeber time; ZT0 corresponds to onset of lights-on; ZT12 corresponds to onset of lights-off). However, in *Bmal1* KO, *Lpin1* continued to oscillate, while *Lipg* was flat.

Summarizing, we found that shared clock-driven mRNA rhythms, which contained core clock and clock-controlled genes, oscillated with significantly larger amplitudes than system-driven genes (Fig. 2G, magenta solid versus dotted). Similarly, clock-driven liver-specific mRNA rhythms also oscillated at higher amplitudes compared with system-driven mRNA rhythms (Fig. 2G, red solid versus dotted). On the other hand, kidney-specific clock- and system-driven transcripts oscillated with comparable amplitudes (Fig. 2G, blue solid versus dotted) and were less numerous overall, which could reflect the distinct cell types constituting the kidney (Lee et al. 2015). The uncovered diversity of clock- and system-driven mRNA rhythms involving distinct combinations of tissues hints at complex transcriptional or post-transcriptional regulation.

### Oscillatory TF activity in one tissue but not others can drive tissue-specific mRNA rhythms

**Figure 3.**
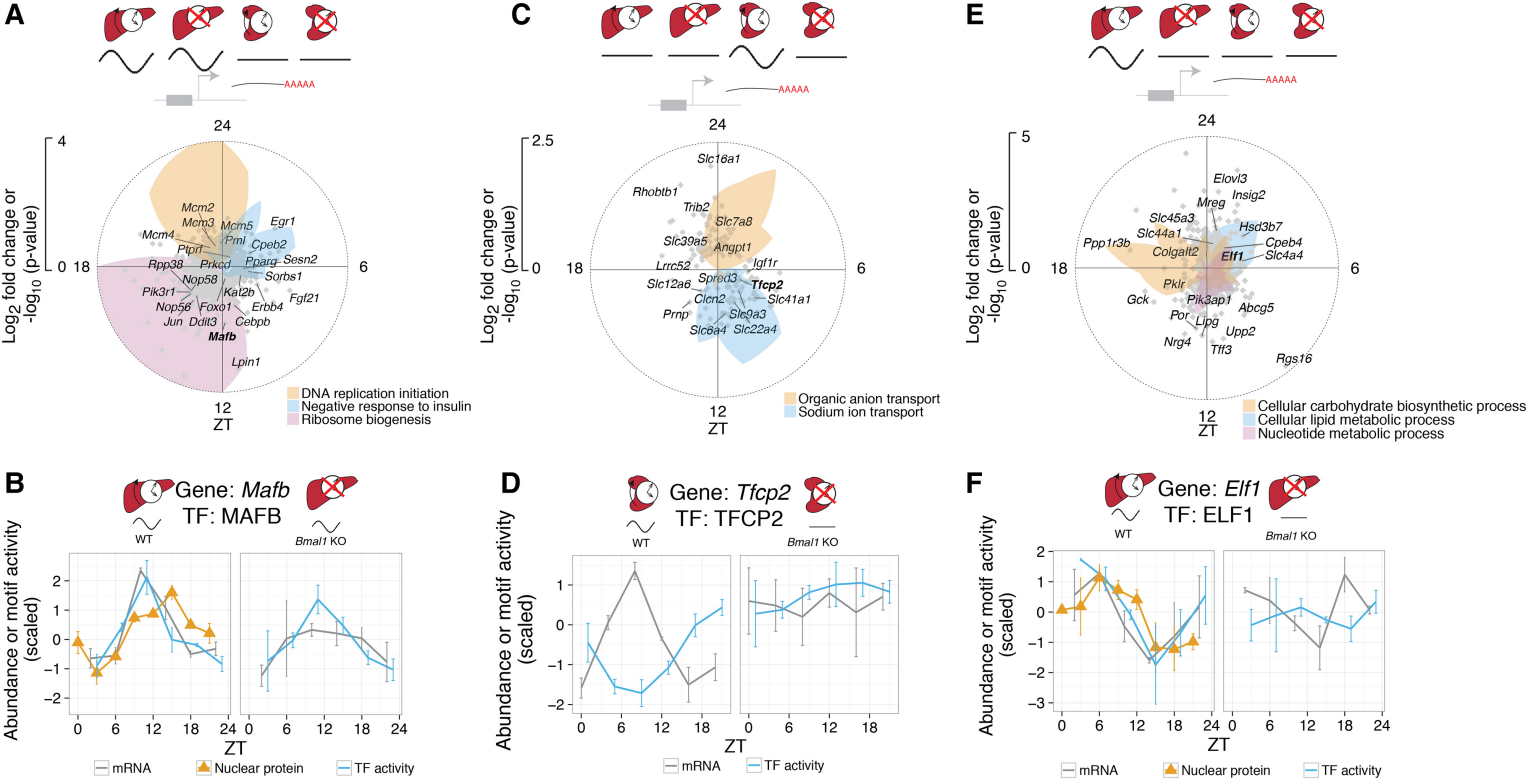
Oscillatory TF activity in one tissue but not others can drive tissue-specific rhythms. (*A*) Module describing system-driven liver-specific rhythms (*n* = 1395, first SVD component explains 84% of variance). Radial coordinate of the colored polygons represents enrichment of the indicated GO terms at each time point, obtained by comparing the genes falling in a sliding window of ±3 h to the background set of all 1395 genes assigned to the module (*P*-value computed from Fisher's exact test). (*B*) MAFB is a candidate TF for the module in *A*. Predicted MAFB activity (blue), nuclear protein abundance (orange triangles), and mRNA accumulation (gray) oscillate in WT and *Bmal1* KO, with peak mRNA preceding peak nuclear protein and TF activity. Error bars in nuclear protein, mRNA, and TF activity show SEM (*n* = 2). (*C*) Clock-driven kidney-specific module (*n* = 156, first SVD component explains 80% of variance). Colored polygons as in *A*. (*D*) TFCP2 is a candidate TF for the module in *C*. The temporal profile of predicted TFCP2 activity (blue) is anti-phasic with *Tfcp2* mRNA accumulation (gray) in WT, and both are flat in *Bmal1* KO. Error bars in mRNA and TF activity show SEM (*n* = 2). (*E*) Clock-driven liver-specific module (*n* = 991, first SVD explains 83% of variance). (*F*) ELF is a candidate TF for the module in *E*. The temporal profile of predicted ELF activity (blue) in WT matches that of nuclear protein abundance in liver (orange triangles), and both are delayed compared to *Elf1* mRNA accumulation (gray). In *Bmal1* KO, ELF activity and *Elf1* mRNA are nonrhythmic. Error bars in nuclear protein, mRNA, and TF activity show SEM (*n* = 2).

We focused on WT and *Bmal1* KO liver and kidney to identify rhythmic TF activities underlying clock- and system-driven tissue-specific mRNA rhythms. We first analyzed liver-rhythmic genes driven by systemic signals (*n* = 1395, MS) (Fig. 3A), which were associated with feeding and fasting rhythms (Gene Ontology analysis around the clock) (Methods). Indeed, ribosome biogenesis was up-regulated most strongly during the first 6 h of the feeding phase (from ZT12 to ZT18) (Jouffe et al. 2013; Chauvin et al. 2014), while insulin signaling was down-regulated during first 6 h of the fasting phase (from ZT0 to ZT6) (Ravnskjaer et al. 2013), consistent with daily responses to nutrient fluctuations in liver (Sinturel et al. 2017).

To infer rhythmic TF activities that may underlie these mRNA rhythms, we applied a penalized regression model (MARA) (Balwierz et al. 2014) that integrates TF binding site predictions near promoters with mRNA accumulation. TF analysis of this module notably identified TFs related to insulin biosynthesis and gluconeogenesis, such as MAFB (Matsuoka et al. 2003) and EGR1 (Matsuoka et al. 2003; Shen et al. 2015), whose activities peaked at ZT11 and ZT3, respectively (Fig. 3B; Supplemental Fig. S4A). Integrating temporal activities of candidate TFs with RNA-seq and our previously described temporal nuclear protein data set (Wang et al. 2017), we found that rhythmic activity of MAFB and EGR1 was supported by rhythmic mRNA abundance, followed by rhythmic nuclear protein abundance (Fig. 3B; Supplemental Fig. S4B), likely reflecting the delayed protein abundance after mRNA accumulation (Mermet et al. 2016).

Next, we analyzed clock-driven transcripts oscillating specifically in the kidney (*n* = 156, MS) (Fig. 3C), among which sodium ion and organic anion transporters peaked near ZT12 and ZT0, respectively. The up-regulation of sodium ion transporters in kidney during the behaviorally active phase may underlie clock-dependent increase of sodium excretion (Nikolaeva et al. 2012). Similarly, the up-regulation of organic anion transporters during the resting phase may explain increased transport activity for precursors of gluconeogenesis, such as pyruvate and lactate, during fasting (Stumvoll et al. 1998; Ekberg et al. 1999). TF analysis predicted TFCP2 to regulate mRNAs that peaked during the resting phase (Fig. 3D; Supplemental Fig. S4C). The predicted TFCP2 activity was anti-phasic with *Tfcp2* mRNA abundance, suggesting a repressive activity, consistent with the ability of TFCP2 to recruit histone deacetylase HDAC1 (Kim et al. 2016).

Finally, liver-specific clock-driven rhythmic transcripts (*n* = 991, MS) were comprised of genes associated with glucose metabolism (enriched at ZT18), such as *Gck* and *Ppp1r3b* (Kelsall et al. 2009; Oosterveer and Schoonjans 2014)*,* as well as lipid, cholesterol, and bile acid metabolism genes (enriched at ZT2), such as *Elovl3, Insig2, Hsd3b7,* and *Cyp8b1* (Fig. 3E; Shea et al. 2007; Le Martelot et al. 2009; Guillou et al. 2010; Sayin et al. 2013). Predicted activity of ELF oscillated and peaked near ZT3 in WT liver but was flat in *Bmal1* KO (Fig. 3F; Supplemental Fig. S4D; Fang et al. 2014). Interestingly, mRNA abundance of *Elf1*, as well as its nuclear protein abundance, also oscillated in WT, supporting *Elf1* as a potential regulator of oscillating transcriptions peaking near ZT6.

### Colocalized binding of clock- and liver-specific TFs drives liver-specific mRNA rhythms

**Figure 4.**
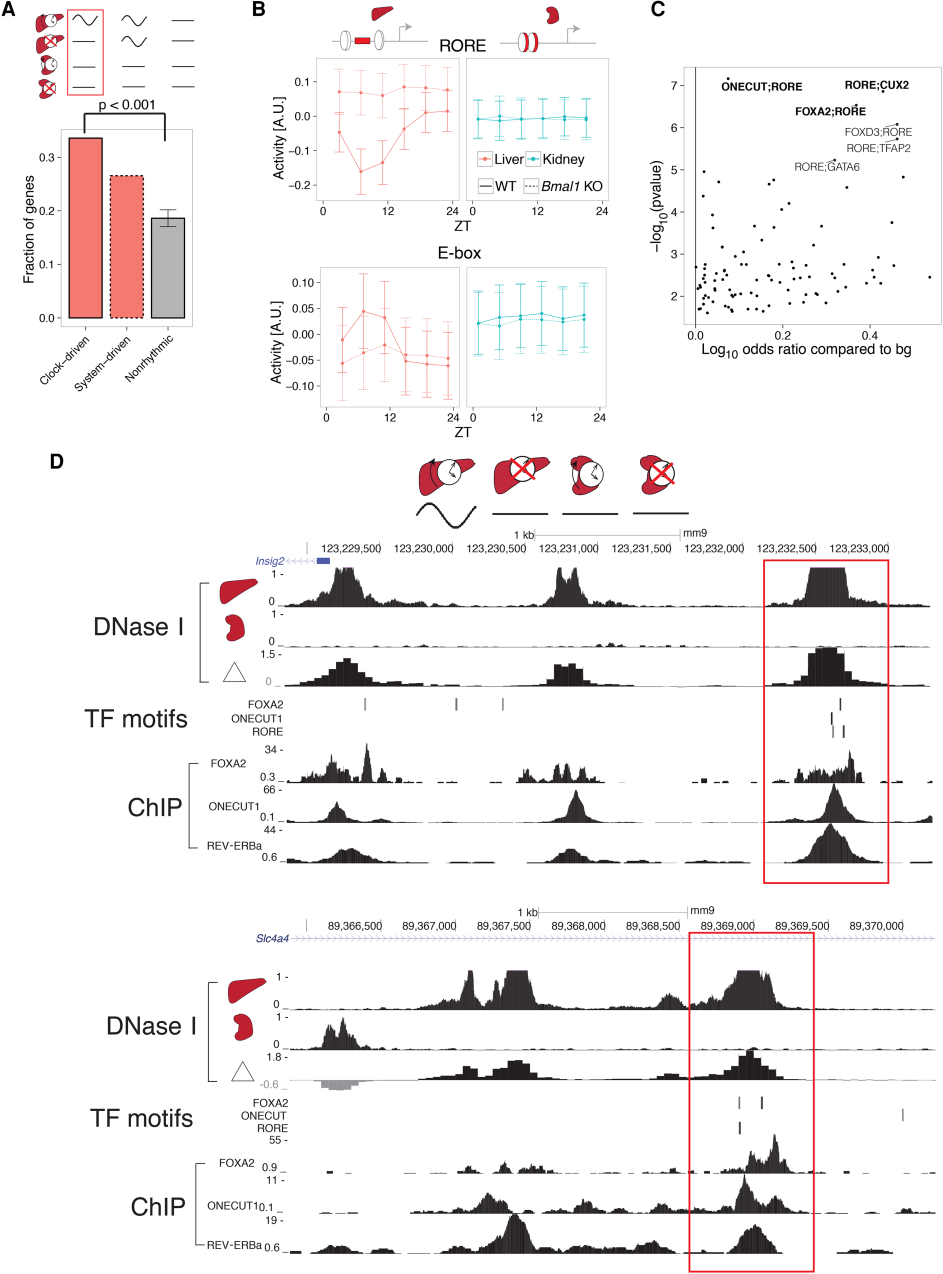
Colocalized binding of clock- and liver-specific TFs underlies liver-specific mRNA rhythms. (*A*) The fraction of genes containing liver-specific DNase I hypersensitive sites (DHSs) in the clock-driven liver-specific module is higher compared with both nonrhythmic and system-driven liver-specific modules. Error bars and *P*-values calculated from 10,000 bootstrap iterations. (*B*) Predicted temporal activities of RORE (*top*) and E-box (*bottom*) TF motifs located within liver-specific DHSs. Error bars show standard deviation of the estimated activities. (*C*) Co-occurrence of RORE with all other TFs in the SwissRegulon database (Pachkov et al. 2007) (189 TF motifs). Positive log_10_ odds ratios (ORs) represent pairs of motifs enriched in the clock-driven liver-specific module compared to the flat module. *P*-values for the motif pairs were calculated from χ^2^ tests applied to three-way contingency tables (Myšičková et al. 2012). Selected pairs are in bold. (*D*) DNase I hypersensitivity in liver, kidney, and the corresponding differential signal (in log_2_ fold change) near two representative genes (*top*: *Insig2*; *bottom*: *Slc4a4*). RORE, ONECUT1, and FOXA2 TF binding motifs (posterior probability > 0.5, MotEvo) co-occur at liver-specific DHSs (red boxes). Predicted TF binding sites correspond to experimentally observed TF binding in publicly available ChIP-exo data sets for REV-ERBa, ONECUT1, and FOXA2 (*bottom*).

To further dissect liver-specific clock-driven rhythms, we reasoned that accessible chromatin regions specific to the liver could harbor regulatory sites for clock TFs, which could then regulate mRNA rhythms liver-specifically. Comparing DNase I hypersensitive sites (DHSs) in liver and kidney (DNase-seq data from ENCODE) (Yue et al. 2014), we found that liver-specific clock-driven genes were enriched with liver-specific DHSs (within 40 kb of promoters), compared to system-driven as well as nonrhythmic genes (Fig. 4A). Using TF binding site predictions underlying these liver-specific DHSs, we applied MARA to predict rhythmic TF activities that explain gene expression of this module (Supplemental Fig. S5A). In WT liver, the predicted activity of RORE oscillated with robust amplitudes and peaked near ZT21. RORE activity became high and flat in *Bmal1* KO liver, consistent with loss of REV-ERB expression and consequently de-repression of REV-ERB target genes (Fig. 4B, top; Bugge et al. 2012). Activity of E-box in WT liver peaked at ZT7, consistent with BMAL1:CLOCK activity (Rey et al. 2011), albeit with weaker amplitudes compared to RORE activity, likely reflecting fewer E-box target genes compared to RORE in this module. In *Bmal1* KO mice, E-box activity was low and flat in liver, as expected.

We hypothesized that cooperativity of liver-specific and clock TFs at liver-specific DHSs can regulate liver-specific mRNA rhythms. Pairwise analysis of TF binding sites at liver-specific DHSs found enrichment of co-occurrence between RORE and liver-specific TF motifs, FOXA2, ONECUT, and CUX2 (Fig. 4C). Enrichment of both CUX2 and ONECUT (also named HNF6) is consistent with ONECUT1 binding to both ONECUT and CUX2 motifs (Conforto et al. 2015). mRNAs of genes with co-occurrence of RORE and liver-specific TF motifs peaked near ZT1, consistent with peak RORE activity (near ZT21) preceding peak mRNA abundance of REV-ERB targets (Supplemental Fig. S5B). Analysis of ChIP-exo data sets targeting FOXA2, ONECUT1, and REV-ERBa in liver (Wang et al. 2014; Zhang et al. 2015; Iwafuchi-Doi et al. 2016) confirmed colocalized TF binding at liver-specific DHSs distal from clock-driven liver mRNAs such as *Insig2* and *Slc4a4* (Fig. 4D).

### Liver-specific chromatin loops regulate liver-specific mRNA rhythms

**Figure 5.**
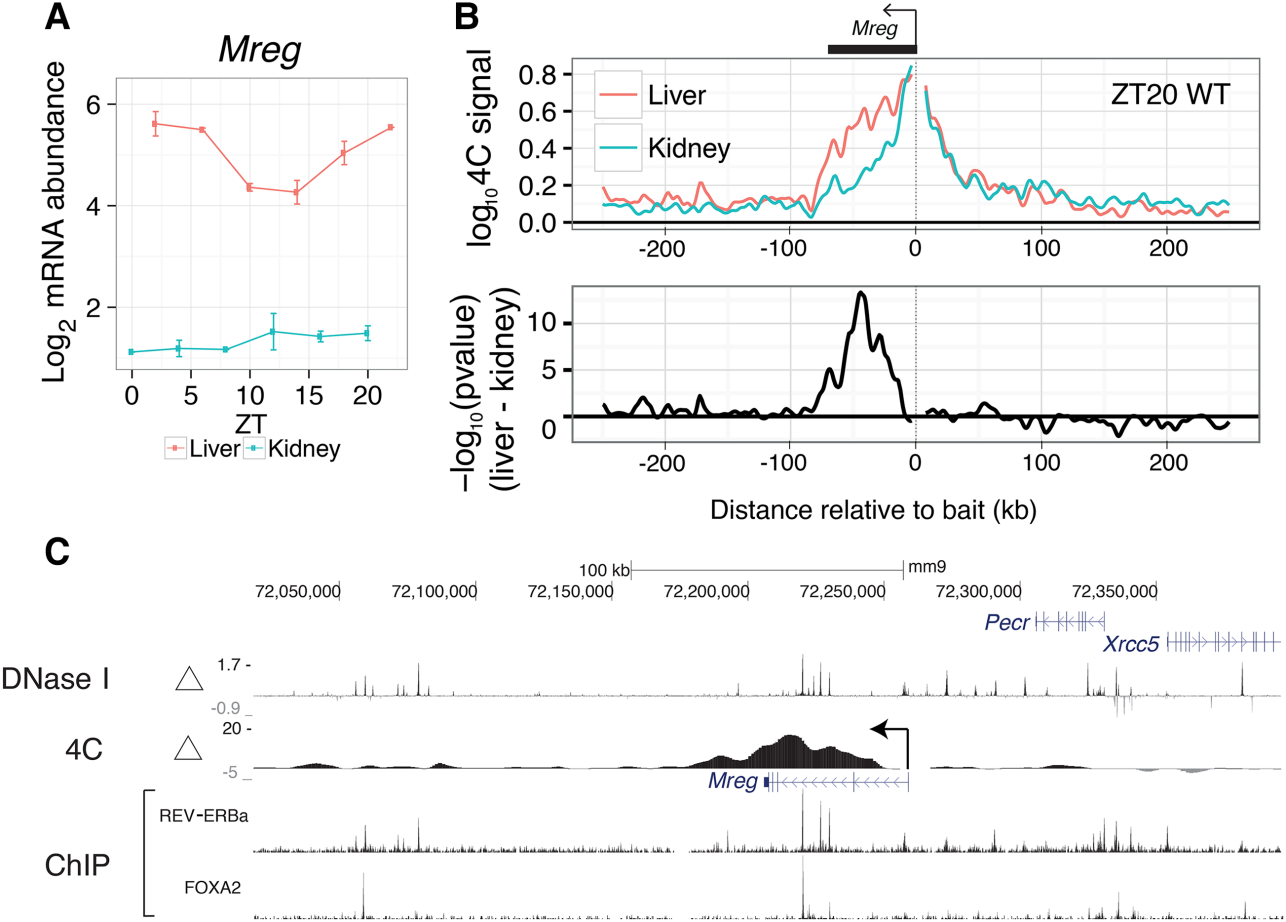
Liver-specific chromatin loops regulate liver-specific mRNA rhythms. (*A*) Temporal mRNA profile for *Mreg*, a clock-driven liver-rhythmic gene. Error bars are SEM (*n* = 2). (*B*) 4C-seq profiles (summary from two replicates, each pooling two different mice) using the *Mreg* promoter as a bait in liver and kidney at ZT20. Data are shown in a window of ±250 kb from the bait (*top*). Profiles of differential contacts between liver and kidney (*bottom*) represented as signed log *P*-values (regularized *t*-test, positive values denote liver-enriched 4C contacts). (*C*) Tracks of differential 4C contacts (signed log *P*-values), log_2_ fold change of DNase I hypersensitivity between liver and kidney, and ChIP-exo of REV-ERBa and FOXA2. Regions of significant differential 4C contacts correspond to liver-specific DNase I hypersensitive regions and REV-ERBa binding sites.

To test whether distally located liver-specific DHSs can contact promoters of clock-driven liver-rhythmic genes, we selected the promoters of *Mreg, Pik3ap1,* and *Slc44a1* as baits for 4C-seq experiments in liver and kidney harvested at the time of peak mRNA accumulation for the selected genes (Methods; Fig. 5A; Supplemental Figs. S6A, S7A). Upstream of *Mreg*, the 4C-seq signal, which measures frequency of promoter-enhancer contacts (van de Werken et al. 2012), decayed rapidly to background level in both liver and kidney (Fig. 5B, top). Downstream from *Mreg*, however, the 4C-seq signal showed a tissue-dependent pattern, decaying slowly in the liver but more rapidly in the kidney. This difference in decay suggests increased frequency of promoter-enhancer contacts in the liver compared to the kidney. Indeed, differential analysis identified liver-specific chromatin contacts 40 kb downstream from the promoter (Fig. 5B, bottom). Overlaying the contact data with DNase-seq, we found that liver-specific chromatin contacts downstream from *Mreg* connected liver-specific DHSs with the *Mreg* promoter (Fig. 5C). Furthermore, ChIP-exo showed colocalization of REV-ERBa and FOXA2 binding at liver-specific DHSs contacting the promoters (Fig. 5C). In contrast, accessible regions upstream of the *Mreg* promoter did not show liver-specific chromatin contacts. The 4C-seq data thus suggest that liver-specific chromatin loops can recruit clock-bound distal elements to promoters to regulate liver-specific transcriptional rhythms. Other liver-specific rhythmic transcripts, *Pik3ap1 and Slc44a1,* also displayed liver-specific chromatin loops between promoter and liver-specific open chromatin regions (Supplemental Figs. S6, S7). In sum, tissue-specific chromatin looping can drive tissue-specific mRNA rhythms.

### Precise promoter-enhancer contacts underlie liver-specific mRNA rhythms

**Figure 6.**
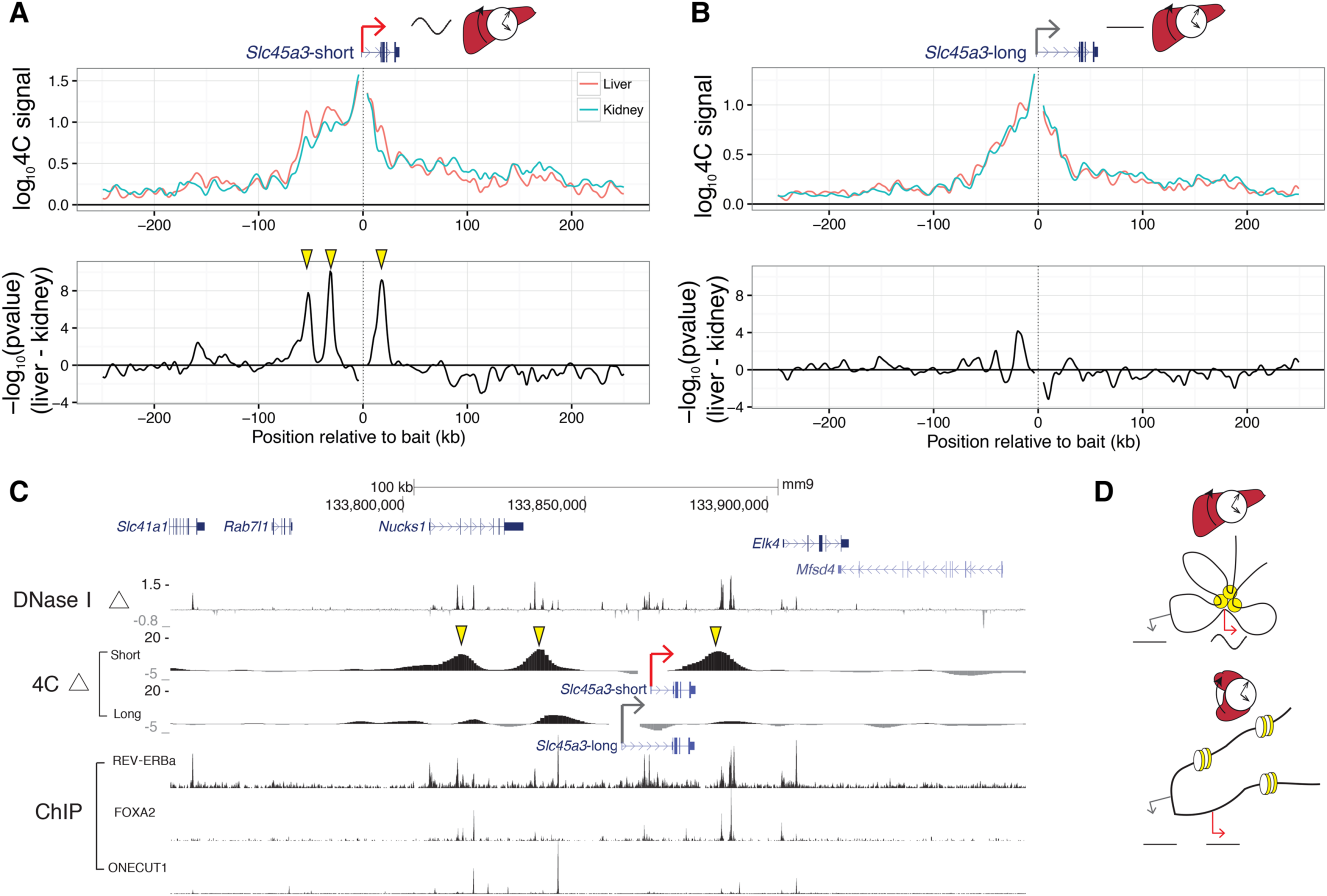
Precise promoter-enhancer contacts underlie liver-specific mRNA rhythms. (*A*,*B*) 4C-seq profiles for the (*A*) *Slc45a3-*short and (*B*) *Slc45a3-*long isoforms within ±250 kb around baits targeting the two TSSs (*top*). Signed log *P*-values for differential contacts between liver and kidney (*bottom*) as in Figure 5B. TSSs for *Slc45a3-*short and *Slc45a3-*long are 8 kb apart. (*C*) Differential 4C contacts (signed log *P*-values), log_2_ fold change of DNase I hypersensitivity between liver and kidney, and ChIP-exo signal of REV-ERBa, FOXA2, and ONECUT1. Regions of significant differential contacts in *Slc45a3-*short correspond to liver-specific DNase I hypersensitive regions. Yellow arrowheads in *A* and *C* show liver-specific distal contacts recruited to the *Slc45a3*-short TSS. These contacts are absent for *Slc45a3*-long TSS (*B*). (*D*) Schematic model illustrating enhancer-promoter interactions in liver and kidney that may generate liver-specific rhythms. Yellow circles illustrate liver-active enhancers contacting the rhythmic promoter (red arrow) but not the alternative nonrhythmic promoter (gray arrow). In kidney, the enhancer is not accessible, and both promoters are nonrhythmic.

To test whether distinct chromatin loops would form at alternative nearby gene promoters with distinct temporal mRNA profiles, we searched for candidate genes where one promoter was rhythmically transcribed while the alternative one was nonrhythmic (Supplemental Fig. S8). *Slc45a3* has two alternative transcripts using different promoters 8 kb apart. The shorter pre-mRNA oscillated in the liver (rhythmic promoter, *Slc45a3-*short), while the longer did not (flat promoter, *Slc45a3-*long). In kidney, neither *Slc45a3-*short nor *Slc45a3-*long showed robust transcript rhythms (Supplemental Fig. S9). Targeting the *Slc45a3-*short promoter with 4C-seq in liver and kidney showed liver-specific chromatin loops at three distal regions (two upstream, one downstream) (Fig. 6A). These same regions did not form liver-specific chromatin loops with the *Slc45a3-*long promoter (Fig. 6B), suggesting that promoters 8 kb apart can contact distinct enhancers. Overlaying 4C-seq with DNase-seq showed that these chromatin loops link liver-specific DHSs specifically to the *Slc45a3-*short promoter (Fig. 6C). These liver-specific DHSs were bound by liver-specific TFs, FOXA2 and ONECUT1, and clock TF, REV-ERBa, as shown in ChIP-seq. The 4C experiments suggest that enhancers can contact a rhythmic promoter while looping out nearby nonrhythmic alternative promoters, confining rhythmic enhancer activity to specific promoters (Fig. 6D). Furthermore, rhythmically active enhancers can contact promoters in a tissue-specific manner. Thus, chromatin folding not only regulates tissue-specific rhythms but also differentiates between closely spaced promoters to control rhythmic transcription with spatial precision.

## Discussion

The mammalian genome encodes transcriptional programs that allow the molecular clock to robustly oscillate across diverse tissue transcriptomes while maintaining flexibility to regulate distinct clock outputs in different combinations of tissues. Here, we identified two regulatory modes underlying tissue-specific transcript rhythms: Regulatory sequences can recruit individual TFs bearing rhythmic activity; coordinated binding of clock- and tissue-specific TFs can generate tissue-specific rhythms. Moreover, we found that clock- and tissue-specific TFs bound at distal enhancers can be recruited to promoters through precise chromatin loops.

Several of our predictions of transcription regulators and regulated genes (e.g., *Egr1*, *Por*, *Upp2*) corroborated with previous analyses of independent data sets (Yan et al. 2008; Bozek et al. 2009; Bhargava et al. 2015). Further analysis incorporating outputs of enhancer activity, such as eRNAs (Fang et al. 2014), across multiple tissues may uncover additional rhythmically active regulators.

Colocalized binding of clock- and tissue-specific TFs at enhancers provides a putative mechanism for the clock to regulate clock output genes in a tissue-specific manner. In mouse liver, clock TFs can colocalize with liver-specific TFs, such as FOXA2 and ONECUT1, consistent with multiple TFs associating with liver-specific DHSs (Iwafuchi-Doi et al. 2016). Our findings are currently based on sequence-specific DNA binding of TFs, comparison of tissues, and ChIP-seq data sets. Further mechanistic basis for the functional significance of colocalization could be gained, for example, by using inducible knockout models for tissue-specific regulators. Moreover, the observed colocalization does not exclude other cooperative modes, such as tethering of REV-ERBa to ONECUT1 through protein-protein interactions (Zhang et al. 2015).

Our 4C analysis showed that chromatin looping might mediate interaction between clock- and tissue-specific transcriptional programs by recruiting clock-bound distal elements to promoters in a tissue-specific manner. Such loops can surgically discriminate between nearby promoters as close as 8 kb apart, suggesting a way to separate temporal regulation of neighboring promoters. A previous 4C study on a core clock gene enhancer proposed that cohesion-mediated promoter-enhancer looping can compartmentalize rhythmic gene expression within genomic regions spanning 150 kb (Xu et al. 2016). Here, chromatin interactions that differed between tissues were localized to a small genomic region (<10 kb) near promoters (<100 kb). Future studies integrating temporal data across tissues with large-scale promoter-enhancer networks may reveal regulatory sequences that encode promoter-enhancer compatibility and elucidate whether this compatibility is tissue-specific (Li and Noll 1994; Merli et al. 1996; Zabidi et al. 2014; Nguyen et al. 2016).

While our work focused on transcriptional mechanisms, studying other mechanisms such as post-transcriptional, translational, and post-translational processes using PRO-seq, Ribo-seq, and proteomics data may provide additional insights. Expanding our 24-h analysis to 12-h or other harmonics would broaden the view of tissue-specific temporal gene expression but may require experimental designs of higher temporal resolution (Hughes et al. 2009; Krishnaiah et al. 2017). In sum, integrating the temporal axis into tissue-specific gene regulation offers an integrated understanding of how tissue physiology resonates with daily cycles in the environment.

## Methods

### Animal experiments

Eight- to 14-wk-old C57Bl/6 mice have been purchased from Charles River Laboratory. *Bmal1* KO mice have been previously described (Jouffe et al. 2013). Without further indications, mice are kept under a 12-h light/12-h dark regimen and ad libitum feeding. All animal care and handling was performed according to the Canton de Vaud (Fred Gachon, authorization no. VD 2720) laws for animal protection.

### RNA-seq experiments

To complement the mouse liver WT and *Bmal1* KO RNA-seq data (GSE73554) (Atger et al. 2015), transcriptomes of kidneys from *Bmal1* KO and WT littermates (12-h light/12-h regimen; night-restricted feeding) were measured from poly(A)-selected mRNA using single-end reads of length 100. mRNA levels were quantified using kallisto version 0.42.4 (mm10) (Bray et al. 2015).

#### Global temporal variance

For each tissue, we estimated the contribution of temporal variance for each gene, broken down by its Fourier components. We calculated the background level assuming temporally unstructured data (white noise), whose magnitude (strength of the white noise) was estimated from the mean of squared magnitudes of Fourier coefficients that were not submultiples of 24 h (i.e., the mean of 48-, 16-, 9.6-, 6.9-, 5.3-, 4.4-h components).

#### Model selection

We fitted harmonic regression models that integrated temporal gene expression across different combinations of rhythms in different conditions (Atger et al. 2015). We used a g-prior for the rhythmic parameters $\vec{\;\beta \;}$ as a penalization scheme (Liang et al. 2008). We set g = 1000, which we found to maximize temporal variations captured in the shared rhythms model while minimizing temporal variations captured in the flat model.

#### Complex singular value decomposition representation of gene and tissue module

We first transformed the time domain to the frequency domain corresponding to 24-h rhythms. The resulting matrix was decomposed using SVD; the first left-singular and first right-singular values were visualized in separate polar plots (Supplemental Methods).

#### Functional analysis by GO terms

We used Fisher's exact test to assess statistical significance of gene enrichment for each GO term. Foreground genes were genes with phases within a 6-h window. Background genes were genes assigned to a model. For each GO term, we slid the 6-h window with a step size of 1 h and calculated the *P*-value enrichment. GO terms were chosen by visualizing significant GO terms in the tree and choosing GO terms that were comparably deep in the tree.

### Chromatin conformation experiments and analysis

C57Bl/6 mice were sacrificed at ZT08 and ZT20 to extract liver and kidneys. Liver and kidney nuclei were prepared as previously described (Ripperger and Schibler 2006) with some minor changes. 4C-seq assays were performed as in Gheldof et al. (2012). Raw read counts for each sample were normalized by library size by the sum of the read counts on the *cis*-chromosome (excluding 10 fragments around the bait). We used a weighted linear model to fit the log_10_ signal around each fragment f. A Gaussian window of standard deviation = 2.5 kb centered on f was used to incorporate signal from neighboring fragments (Supplemental Methods). Differential contacts were estimated using *t*-statistics.

## Data access

Raw and processed RNA-seq and 4C-seq data generated from this study have been submitted to the NCBI Gene Expression Omnibus (GEO; http://www.ncbi.nlm.nih.gov/geo/) under accession number GSE100457.

## Competing interest statement

C.J., J. Marquis, A.C., and F.G. are employees of Nestlé Institute of Health Sciences SA.

## Supplementary Material

Supplemental Material
