# Supplementary material for: Transcription factor activity rhythms and tissue-specific chromatin interactions explain circadian gene expression across organs
Source: Genome Res. 2018 Feb;28(2):182–91. doi: 10.1101/gr.222430.117 (PMC5793782; doi:10.1101/gr.222430.117)
Supplement: Supplemental Material [file supp_28_2_182__index.html]

Transcription factor activity rhythms and tissue-specific chromatin interactions explain circadian gene expression across organs — Supplemental Material 

# Transcription factor activity rhythms and tissue-specific chromatin interactions explain circadian gene expression across organs

## Supplemental Material

- Supplemental\_Fig\_S1.pdf
- Supplemental\_Fig\_S2.pdf
- Supplemental\_Fig\_S3.pdf
- Supplemental\_Fig\_S4.pdf
- Supplemental\_Fig\_S5.pdf
- Supplemental\_Fig\_S6.pdf
- Supplemental\_Fig\_S7.pdf
- Supplemental\_Fig\_S8.pdf
- Supplemental\_Fig\_S9.pdf
- Supplemental\_Methods\_final\_v03.pdf
- Supplemental\_Table\_1\_Hogenesch-Microarray-RNASeq-Merged-Wide.txt.zip
- Supplemental\_Table\_2\_Liver-Kidney-WTKO-RNASeq.txt.zip
- Supplemental\_Table\_3\_Hogenesch-Model-Selection.txt.zip
- Supplemental\_Table\_4\_LivKidWTKO-Model-Selection.txt.zip
