# Supplementary material for: Transcription factor activity rhythms and tissue-specific chromatin interactions explain circadian gene expression across organs
Source: Genome Res. 2018 Feb;28(2):182–91. doi: 10.1101/gr.222430.117 (PMC5793782; doi:10.1101/gr.222430.117)
Supplement: Supplemental Material [file supp_gr.222430.117_Supplemental_Fig_S1.pdf]

Supplemental Figure S1

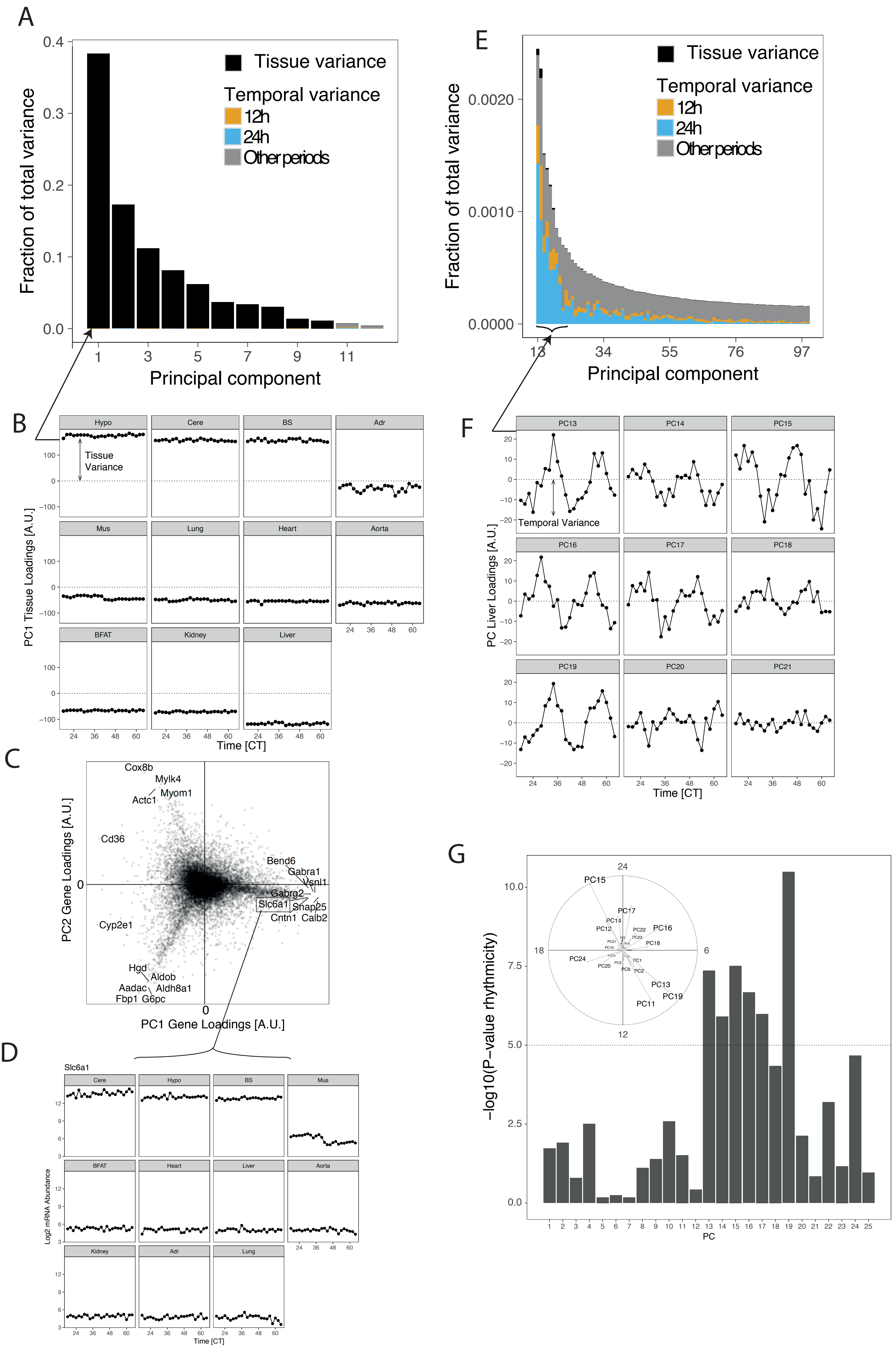

Supplemental Figure S1 - Contribution of tissue and temporal variance across 11 tissues

(A) Fraction of total variance explained by principal components 1 to 12. Colors represent the contribution of different temporal periods (Fourier coefficients) in each principal component. Components 1 to 12 show predominantly tissue differences.

(B) Sample loadings of 11 tissues over time in PC1. Contribution of tissue variance to the principal component is calculated by the sum of the squared difference between tissue mean and the global mean. Variance in PC1 consists of mostly tissue variance.

(C) Scatterplot of gene loadings from PC1 and PC2. Genes with large PC1 loadings are mainly brain-specific genes.

(D) mRNA abundance across tissues and time of a brain-specific GABA transporter, *Slc6a1*.

(E) *idem* as A, for principal components 13 to 100.

(F) Liver loadings over time for PCs 13 to 21. Contribution of temporal variance to the principal component is calculated by the sum of the squared difference between each time point and the tissue mean.

(G) Analysis of 24h rhythmicity of liver loadings over time (PC1 to PC25, harmonic regression). Inset: Amplitude (radial coordinate) and phase (clockwise angle) of liver loadings in PC1 and PC25.
