# Supplementary material for: Transcription factor activity rhythms and tissue-specific chromatin interactions explain circadian gene expression across organs
Source: Genome Res. 2018 Feb;28(2):182–91. doi: 10.1101/gr.222430.117 (PMC5793782; doi:10.1101/gr.222430.117)
Supplement: Supplemental Material [file supp_gr.222430.117_Supplemental_Fig_S2.pdf]

Supplemental Figure S2

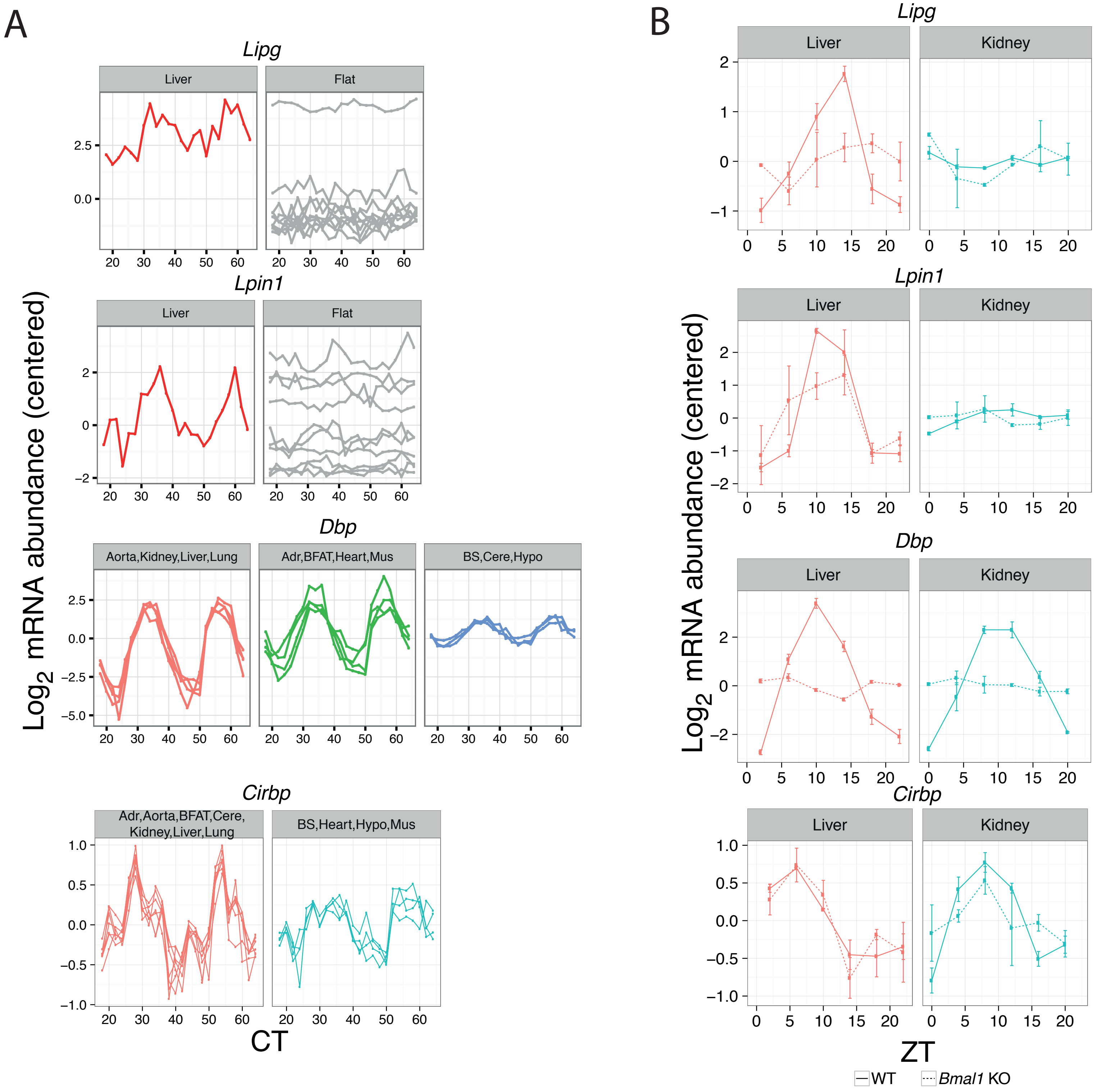

Supplemental Figure S2 – Temporal profiles of mRNA accumulation across tissues and in clock-disrupted conditions

(A) Examples of genes whose expression is rhythmic in different combinations of tissues. Flat indicates nonrhythmic tissues. Rhythms can be further classified as clock- or system-driven by analysis in WT and *Bmal1* KO data, shown in (B).

(B) Examples of tissue-specific (*Lipg, Lpin1*) and tissue-wide (*Dbp, Cirbp*) genes whose oscillations can be clock-dependent (*Lipg, Dbp*) or –independent (*Lpin1, Cirbp*) manner.
