# Supplementary material for: Transcription factor activity rhythms and tissue-specific chromatin interactions explain circadian gene expression across organs
Source: Genome Res. 2018 Feb;28(2):182–91. doi: 10.1101/gr.222430.117 (PMC5793782; doi:10.1101/gr.222430.117)
Supplement: Supplemental Material [file supp_gr.222430.117_Supplemental_Fig_S3.pdf]

Supplemental Figure S3

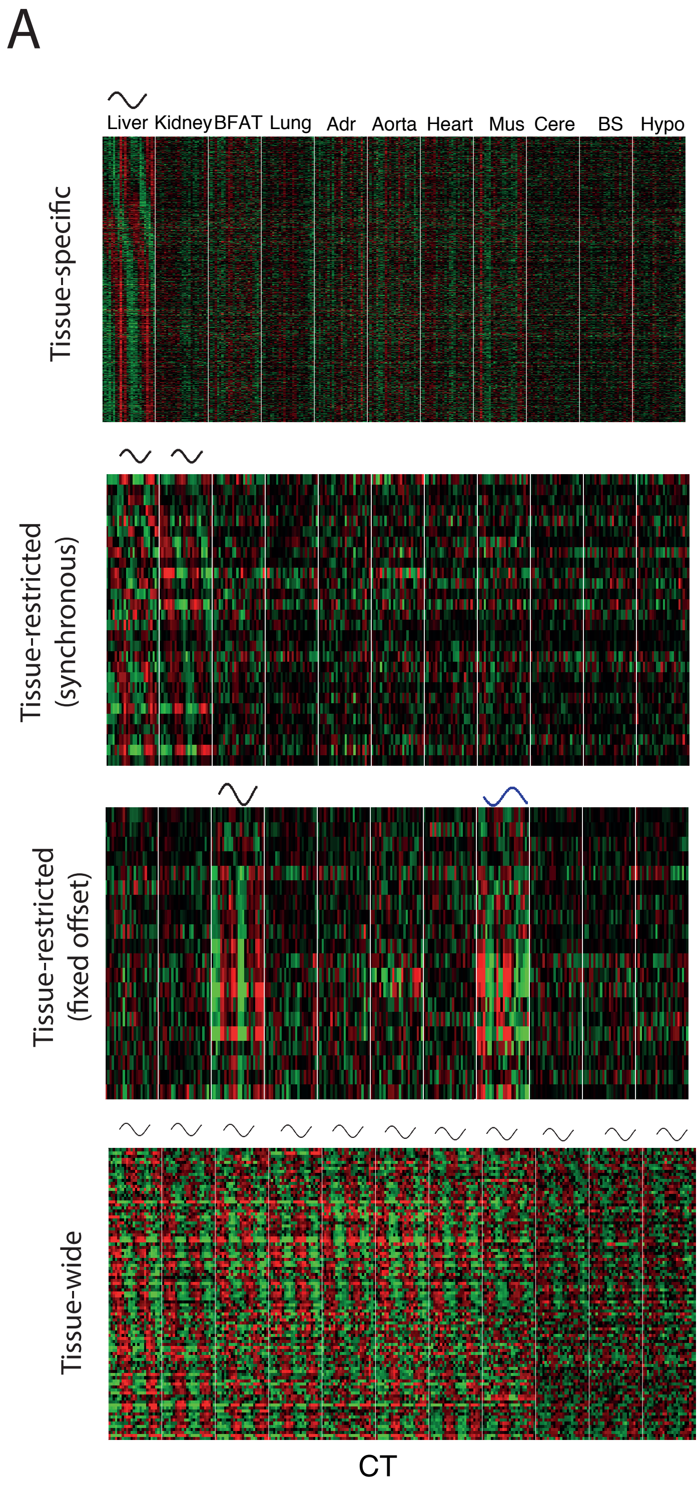

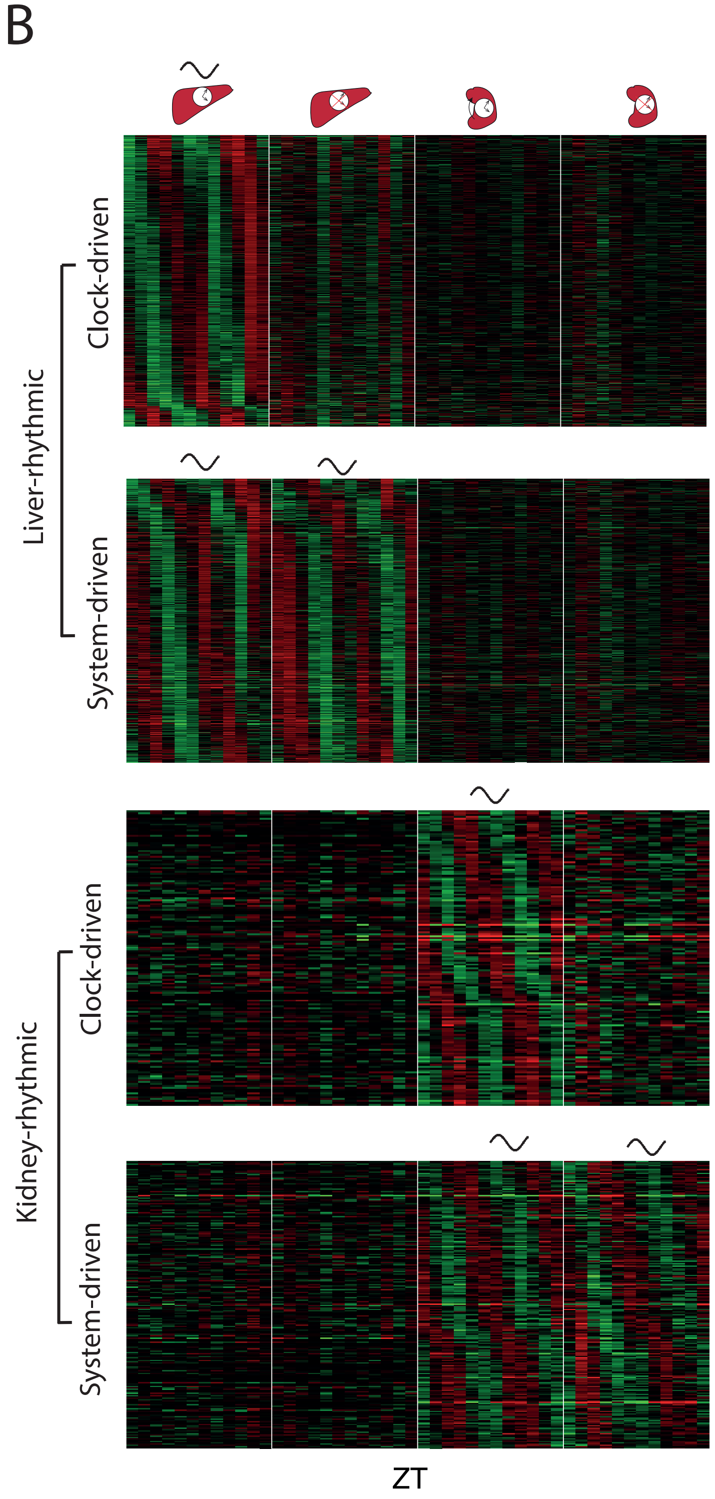

Supplemental Figure S3 – Modules of rhythmic gene expression across different subsets of tissues

(A) Heatmaps for the modules corresponding to genes rhythmic in different subsets of tissues. Each rectangle, demarcated by white vertical lines, represents normalized mRNA accumulation over 48 hours (red denotes low expression, green denotes high) for each gene in module (y-axis) in a single tissue. Schematic of the combination of tissues in which genes are rhythmic are shown above each heatmap. For clarity, genes in tissue-wide module (bottom) are filtered for amplitudes with average log2 fold change greater than 0.8.

(B) Heatmaps representing modules of liver- and kidney-rhythmic genes that are driven by the local clock or systemic cues.
