# Supplementary material for: Transcription factor activity rhythms and tissue-specific chromatin interactions explain circadian gene expression across organs
Source: Genome Res. 2018 Feb;28(2):182–91. doi: 10.1101/gr.222430.117 (PMC5793782; doi:10.1101/gr.222430.117)
Supplement: Supplemental Material [file supp_gr.222430.117_Supplemental_Fig_S4.pdf]

Supplemental Figure S4

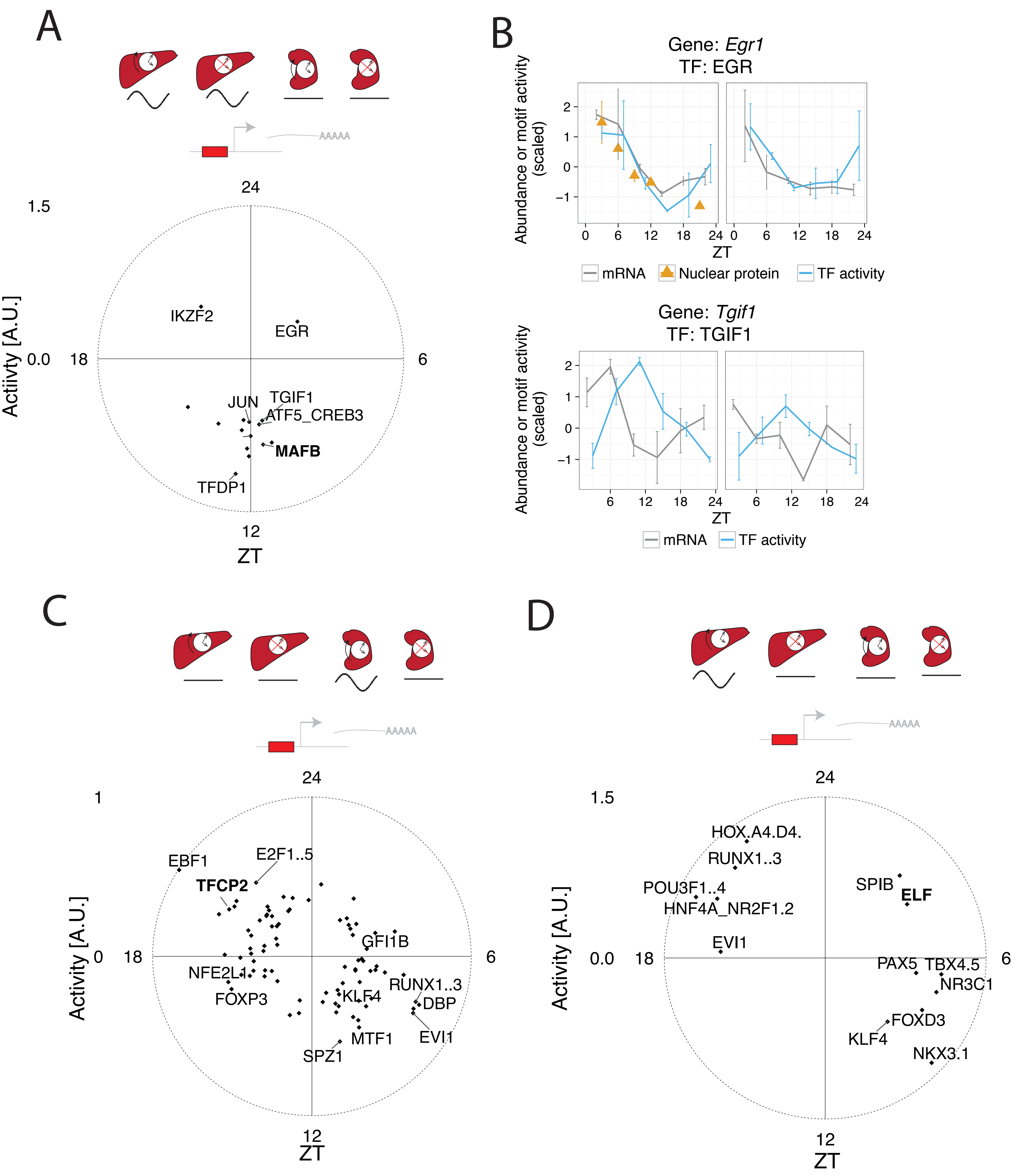

Supplemental Figure S4 - TF regulators associated with tissue-specific rhythmic gene expression

(A) Predicted activities of TF motifs associated with system-driven liver-rhythmic module (left).

(B) Predicted TF activity (solid line), nuclear protein abundance (if available, triangles), and mRNA accumulation (dotted) oscillate in both WT and *Bmal1* KO. Error bars in nuclear protein, mRNA, and TF activity are SEM (n=2).

(C,D) Predicted activities of TF motifs associated with clock-driven kidney-rhythmic (C), and clock-driven liver-rhythmic (D) module represented by the first component of complex-valued SVD. Motifs with z-score > 1.25 are shown. Candidate TFs shown in Figure 4B,D,F are displayed in bold.
