# Supplementary material for: Transcription factor activity rhythms and tissue-specific chromatin interactions explain circadian gene expression across organs
Source: Genome Res. 2018 Feb;28(2):182–91. doi: 10.1101/gr.222430.117 (PMC5793782; doi:10.1101/gr.222430.117)
Supplement: Supplemental Material [file supp_gr.222430.117_Supplemental_Fig_S5.pdf]

Supplemental Figure S5

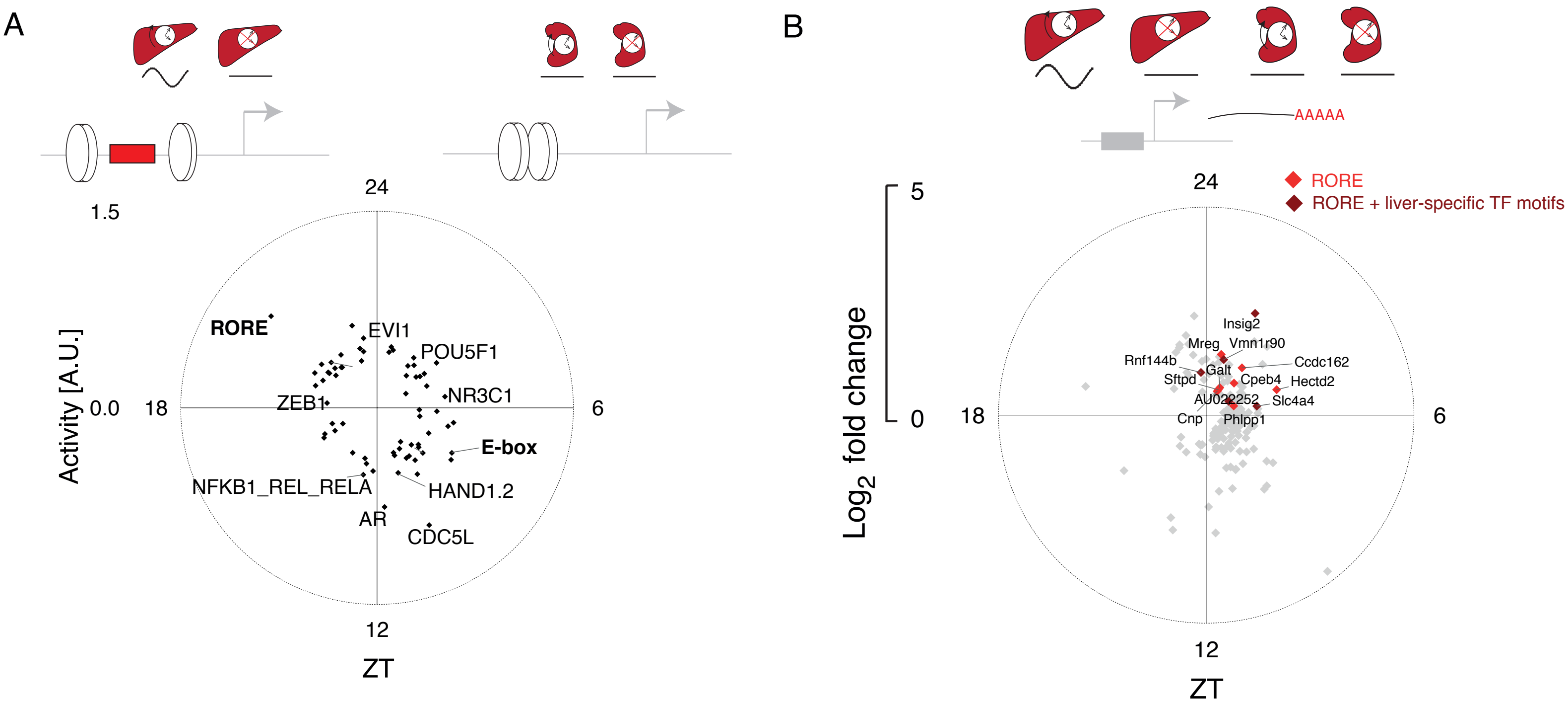

Supplemental Figure S5 – Liver-specific accessible regions harboring clock TF binding sites underlie clock-driven liver-specific rhythms

(A) Activities of TF motifs associated with clock-driven liver-rhythmic module, predicted using TF binding site occurrences at liver-specific DHSs.

(B) Genes containing RORE (red) or co-occurrence of RORE and liver-specific TF (ONECUT1, CUX2, or FOXA2, dark red) in clock-driven liver-rhythmic module. mRNA abundances of genes with liver-specific DHSs harboring RORE motifs peak approximately 3 hours after peak RORE activity.
