# Supplementary material for: Transcription factor activity rhythms and tissue-specific chromatin interactions explain circadian gene expression across organs
Source: Genome Res. 2018 Feb;28(2):182–91. doi: 10.1101/gr.222430.117 (PMC5793782; doi:10.1101/gr.222430.117)
Supplement: Supplemental Material [file supp_gr.222430.117_Supplemental_Fig_S6.pdf]

Supplemental Figure S6

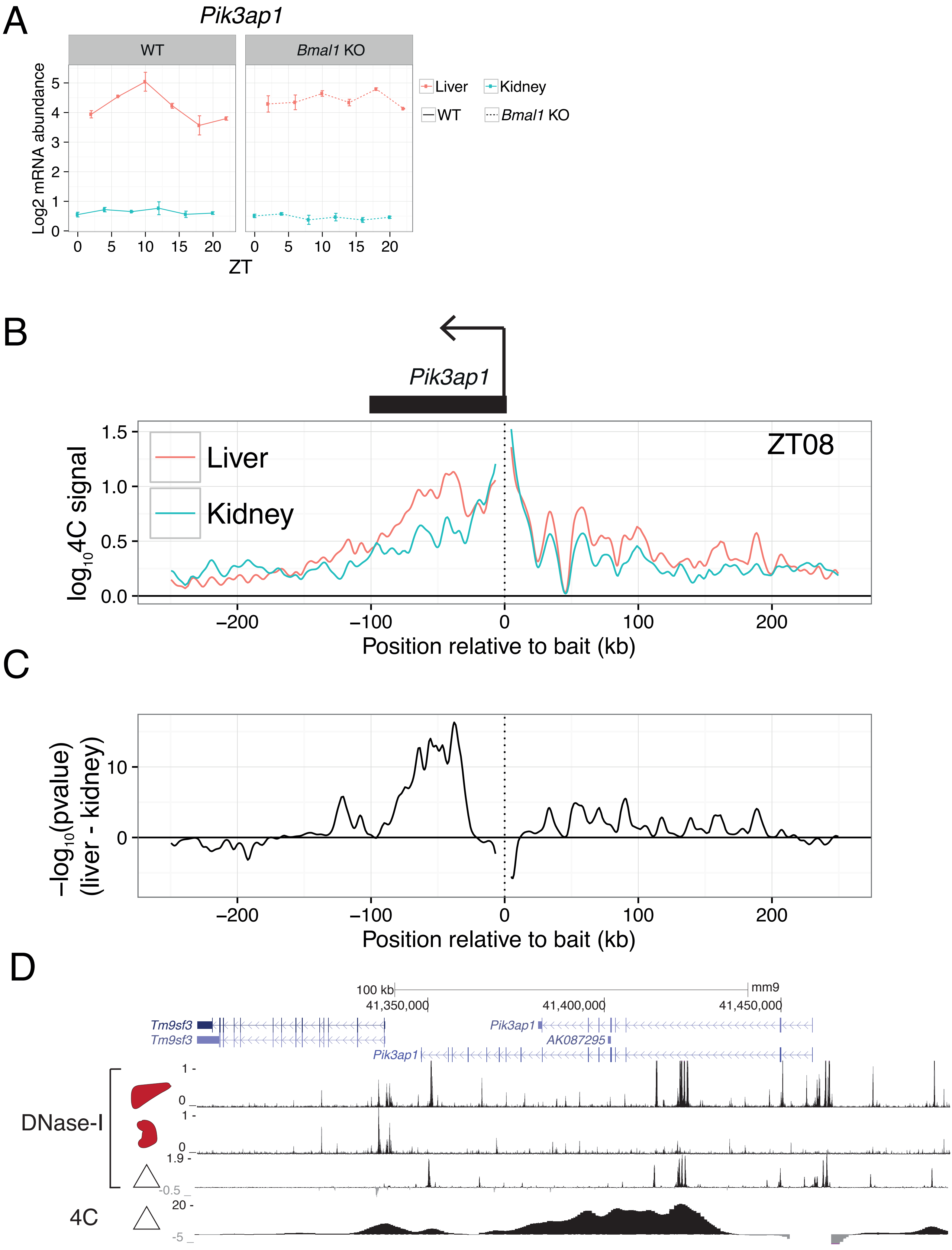

Supplemental Figure S6 - Chromatin loops between liver-specific enhancers and promoter of *Pik3ap1*

(A) Temporal mRNA abundance profiles of *Pik3ap1* in liver and kidney of mice with (left) and without (right) a functioning clock. Nuclei were extracted from WT liver and kidney from 4 mice at ZT8 to perform 4C-Seq.

(B) 4C-Seq profiles (summary from 2 samples, each pooled from 2 mice) using the *Pi3kap1* promoter as a bait in liver and kidney within a window of 500 kb.

(C) Profiles of differential contacts for liver versus kidney, shown as signed log p-values (regularized t-test, positive values show liver-enriched 4C contacts).

(D) Tracks of differential contacts (signed log p-values), DNAse-I hypersensitivity in liver, kidney, and their log2 fold change. Regions of significant differential contacts correspond to liver-specific DHS regions.
