# Supplementary material for: Transcription factor activity rhythms and tissue-specific chromatin interactions explain circadian gene expression across organs
Source: Genome Res. 2018 Feb;28(2):182–91. doi: 10.1101/gr.222430.117 (PMC5793782; doi:10.1101/gr.222430.117)
Supplement: Supplemental Material [file supp_gr.222430.117_Supplemental_Fig_S8.pdf]

Supplemental Figure S8

A

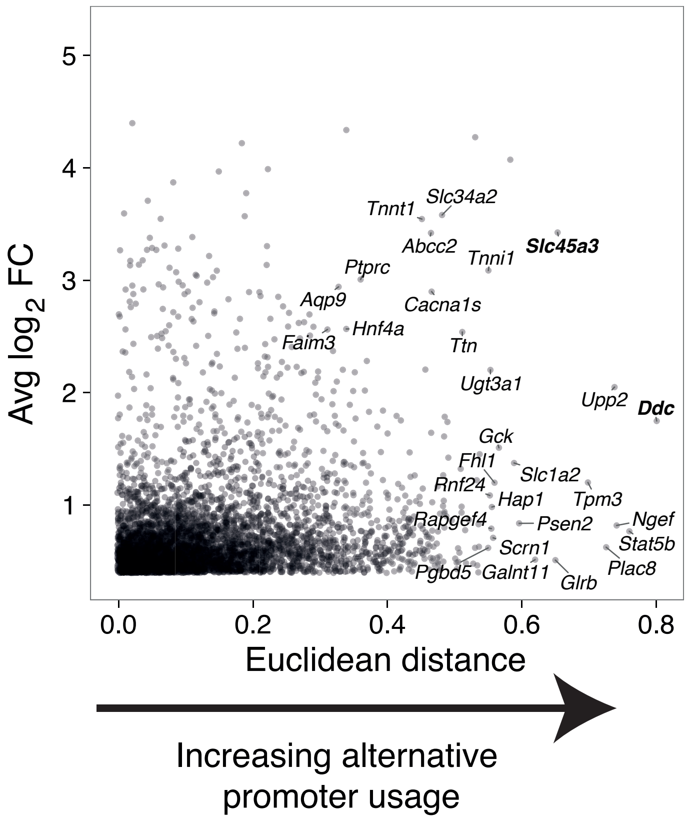

B

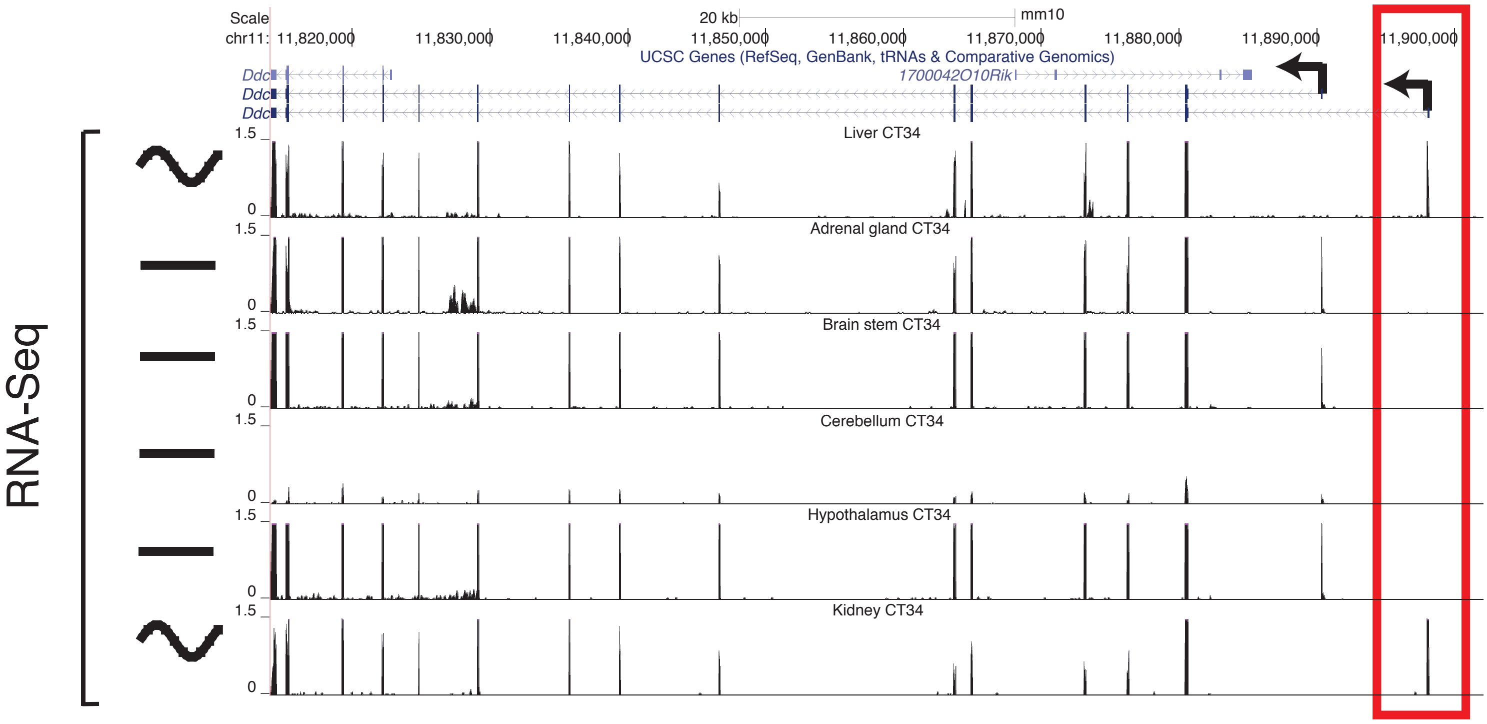

Supplemental Figure S8 - Correlations of alternative transcript start site (TSS) usage and tissue-specific rhythms in mRNA accumulation

(A) Scatterplot of alternative promoter usage versus log2 fold-change (FC) of rhythmic tissues. Alternative TSS usage defined by calculating the Euclidean distance of transcript expression in tissues with rhythmic transcript versus tissues with nonrhythmic transcripts.

(B) Example of alternative TSS usage in rhythmic gene expression. RNA-Seq of *Ddc* at CT34 shows that rhythmic tissues (liver and kidney) use an upstream promoter whereas nonrhythmic tissues use a downstream promoter.
