# Supplementary material for: Transcription factor activity rhythms and tissue-specific chromatin interactions explain circadian gene expression across organs
Source: Genome Res. 2018 Feb;28(2):182–91. doi: 10.1101/gr.222430.117 (PMC5793782; doi:10.1101/gr.222430.117)
Supplement: Supplemental Material [file supp_gr.222430.117_Supplemental_Fig_S9.pdf]

Supplemental Figure S9

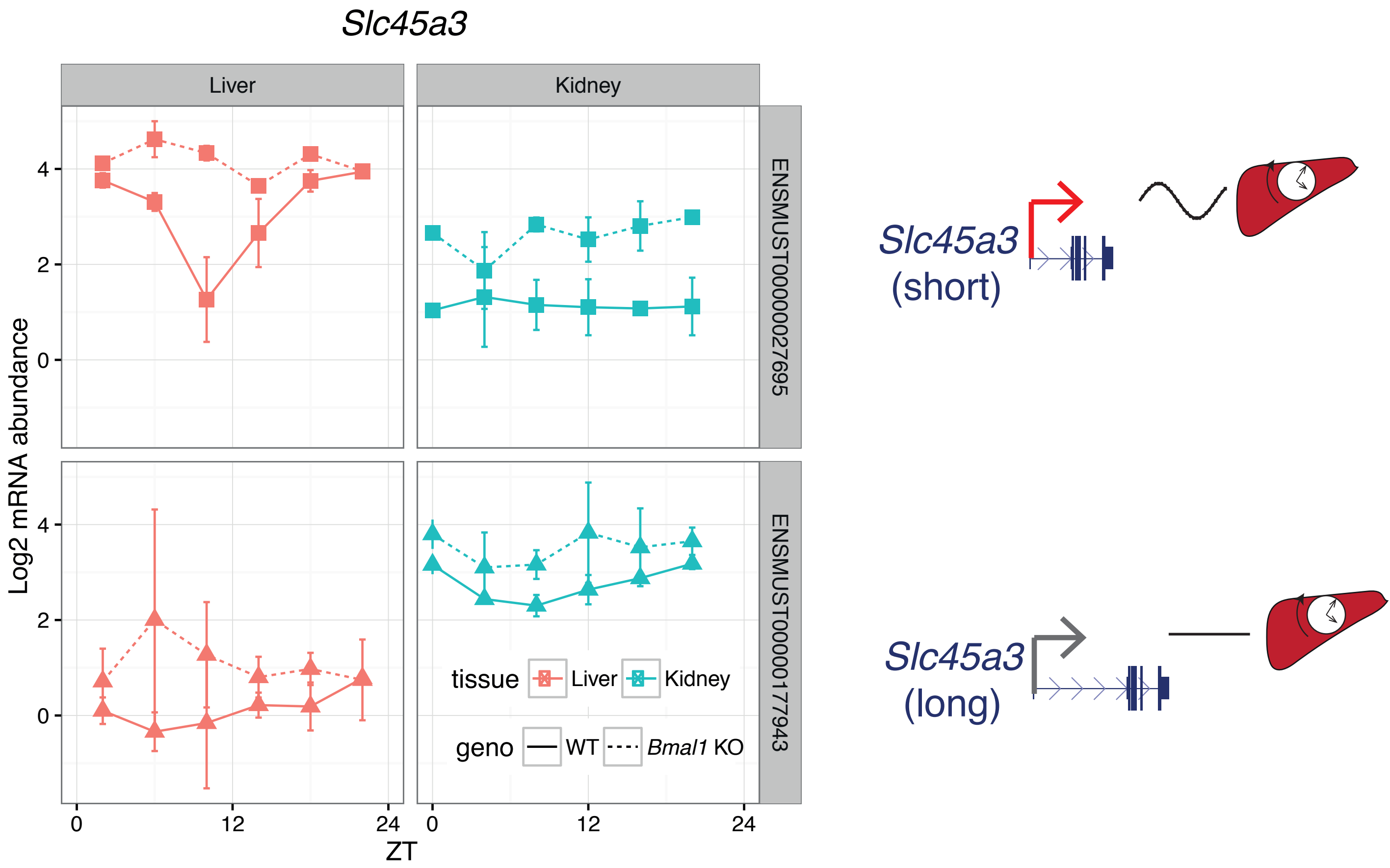

Supplemental Figure S9 — Liver and kidney use different TSSs in *Slc45a3,* a clock-driven liver transcript

Temporal abundance profiles of two transcripts of *Slc45a3* in liver and kidney of mice with and without a functioning clock. *Slc45a3*-short isoform is rhythmic specifically in the liver; *Slc5a3*-long isoform is not robustly rhythmic in liver or kidney.
