# Supplementary material for: Transcription factor activity rhythms and tissue-specific chromatin interactions explain circadian gene expression across organs
Source: Genome Res. 2018 Feb;28(2):182–91. doi: 10.1101/gr.222430.117 (PMC5793782; doi:10.1101/gr.222430.117)
Supplement: Supplemental Material [file supp_gr.222430.117_Supplemental_Methods_.pdf]

Supplemental Materials and Methods

Animal experiments

8-14 weeks old C57Bl/6 mice have been purchased from Charles River Laboratory. *Bmal1* KO mice have been previously described (Jouffe et al. 2013). Without further indications, mice are kept under 12 hours light/12 hours dark regimen and *ad libitum* feeding. All animal care and handling was performed according to the Canton de Vaud (Fred Gachon, authorization no VD 2720) laws for animal protection.

RNA-seq experiments

To complement the mouse liver WT and Bmal1 KO RNA-seq data (GSE73554) (Atger et al. 2015), transcriptomes of kidneys from Bmal1 KO and WT littermates (12 hours light/12 hours regimen; night-restricted feeding) were measured from polyA-selected mRNA using single-end reads of length 100. mRNA levels were quantified using kallisto version 0.42.4 (mm10) (Bray et al. 2015) .

Chromatin conformation experiments

Liver and kidney extraction

C57Bl/6 mice were sacrificed at ZT08 and ZT20 to extract liver and kidneys. Liver and kidney nuclei were prepared as previously described (Ripperger and Schibler 2006) with some minor changes. In brief, liver and kidneys from individual animals were homogenized and fixed in 4ml of PBS including 1.5 % formaldehyde for 10 minutes at room temperature. Cross-linking reaction was stopped by adding 25 ml of ice-cold stop reaction buffer (2.2 M sucrose; 150 mM glycine; 10 mM HEPES pH 7.6; 15 mM KCl; 2 mM EDTA; 0.15 mM spermine; 0.5 mM spermidine; 0.5 mM DTT; 0.5 mM PMSF) to the homogenates and kept 5 minutes on ice. Homogenates were then loaded on top of 10 ml cushion buffer (2.05 M sucrose; 10 % glycerol; 125 mM glycine; 10 mM HEPES pH 7.6; 15 mM KCl; 2 mM EDTA; 0.15 mM spermine; 0.5 mM spermidine; 0.5 mM DTT; 0.5 mM PMSF) and centrifuged for 45 minutes at 10^5 g at 4°C. Nuclei were washed twice in PBS and immediately frozen.

4C-sequencing assay

4C-seq assays were performed as in (Gheldof et al. 2012). Nuclei were resuspended in 1 ml of a buffer containing 10 mM Tris-HCL pH 8.0; 10 mM NaCl; 0.2 % NP-40; 1X protease inhibitor cocktail, (cOmplete, Mini, EDTA-free Protease Inhibitor Cocktail, Sigma-Aldrich) kept for 15 minutes on ice and washed twice with 1X DpnII buffer (New England Biolabs). Approximately 30-million nuclei were resuspended in 1X DpnII buffer (New England Biolabs) containing 0.1 % SDS and incubated at 65°C for 10 minutes. Triton X-100 was added to 1 % final concentration. Chromatin was digested overnight with 400 U DpnII (New England Biolabs) at 37°C with shaking. Digestion was inactivated by incubation at 65°C for 30 minutes in presence of 2 % SDS (final concentration). Digestion efficiency was evaluated both by DNA visualization on agarose gel and by quantitative PCR using primer pairs covering multiple restriction sites (Hagège et al. 2007). Samples presenting reasonable digestion efficiency (greater than 65 %) were then ligated with 3000 U of T4 DNA ligase (New England Biolabs) in 8 ml final volume for 4 hours at 16°C, plus 1 hour at room temperature. Cross-linking reaction was reverted by addition of 50

µl (10 mg/ml) proteinase K and incubation over-night at 65°C. DNA was purified by multiple phenol/chloroform extractions and resuspended in TE buffer pH 8.0 containing RNase A and incubated 30 minutes at 37°C. Ligation efficiency was evaluated by loading DNA ligated products on an agarose gel. Libraries were digested with NlaIII (New England Biolabs) overnight at 37°C and digestion controlled by visualization on an agarose gel. After heat inactivation, digested products were ligated with 2000 U T4 DNA ligase (New England Biolabs) for 4 hours at 16°C in 14 ml final volume. Circularized products were purified and resuspended in TE buffer pH 8.0. 600 ng 4C template was used for PCR amplification using Sigma-Aldrich Long Template PCR System with bait specific inverse primers conjugated to Illumina sequencing adaptors (primer sequences listed in Supplemental Table S5a) in a final volume of 50 µl in the following PCR program: 2 min 94°C, followed by 30 cycles of 15 sec at 94°C, 1 min at 55°C, 3 min at 68°C, final extension 7 min at 68°C. PCR were performed in parallel reactions as 6 x 100 ng template for each sample. PCR products were purified with AMPure XP beads system (Beckman Coulter) and amplification profiles analysed by fragment-analyser and then sequenced on Illumina HiSeq 2000 machines using single end 100 bp read length. PCR products from 2 animals were pooled together, resulting in the sequencing of 2 pooled samples.

Mapping of 4C-seq data

4C-seq data were demultiplexed and mapped to the mouse genome (mm9) using HTS station (http://htsstation.epfl.ch).

Normalization and locally weighted linear regression

Raw read counts for each sample were normalized by library size by the sum of the read counts on the *cis*-chromosome (excluding 10 fragments around the bait). Read counts were log-transformed using the formula:

$$Y = \log_{10}\left(\frac{c}{p} + 1\right)$$

where p=500, the pseudocount.

A weighted linear model was then fit locally, using a Gaussian window ($\sigma_G = 2500$ bp) centered on the fragment of interest. For each position, nearby 4C-seq signals (Y) were modeled with fragment effects a_i and condition effects b_j (which can be time, tissue, or genotype). In LWMR, these parameters are estimated by minimizing the weighted sum S of squared residuals across replicates r:

$S = \arg\min_{a,b} \sum_{i,j,r} W_{i,j}\left(Y_{i,j,r} - a_i - b_j\right)^2$, with weights $W_{i,j}$ are defined as $W_{i,j} = w_{g,i} \times w_{s,j}$, where $w_{g,i}$ is the Gaussian smoothing kernel at position i, and $w_{S,j}$ a condition weight based on the number of samples with non-zero counts on fragment i. Specifically, we used $w_s = (0.5, 1.5, 2.5)$ for fragments with $(0, 1, 2)$ replicates showing non-zero counts, which down weighs positions with high dropout rates.

To estimate statistically significance for differential contacts (liver vs kidney), we propagated the estimated uncertainty (standard errors for locally weighted regression) in the corresponding b's to calculate Z-scores, and used regularized t-statistics with N –

p, degrees of freedom (DOF, N is number of data points within window, p is number of parameters). For each set of samples, we computed the regularized residual variance as $\tilde{\sigma}^2 = \hat{\sigma}^2 + \sigma^2_{\min} \exp\left(-\frac{\bar{b}}{b_s}\right)$ with $\hat{\sigma}^2$ the estimator of the squared residuals, and $\bar{b}$ the estimated signal across samples and $b_s = \log_{10}(2)$. $\sigma^2_{\min}$ prevents artificially small variance from positions of high dropout rates, and is estimated from the distribution of $\hat{\sigma}^2$ across all fragments. $\sigma_{\min}$ was 0.08, 0.02, 0.015, 0.018, and 0.006 for *Mreg*, *Slc44a1*, *Pik3ap1*, *Slc45a3*-short, and *Slc45a3*-long, respectively (same units as Y).

Analysis of Publicly Available Datasets

Atlas of Circadian Gene Expression

Raw Processing

Microarray .CEL files and RNA-seq fastq files were downloaded from Gene Expression Omnibus database (GSE54652) (Zhang et al. 2014). Microarray data were processed using the RMA method implemented by the oligo R package (Release 3.4) with background correction and quantile normalization (Carvalho and Irizarry 2010). RNA-seq fastq files were mapped to the mouse genome (mm10) using STAR (Version 2.4.0) and normalized counts were calculated using DESeq2 (Dobin et al. 2013; Love, Huber, and Anders 2014). Quantification of transcript-level expression was calculated using kallisto (version 0.42.4) (Bray et al. 2015). We removed WFAT from downstream analysis due to concerns with contamination with adjacent epididymal tissue because GO analysis of variable genes in WFAT showed enrichment of epididymal tissue function and this temporal variance did not have rhythms of 24 hours of in other harmonics.

Merging Microarray and RNA-seq

Since microarray data and RNA-seq data came from the same biological samples, we integrated the microarray and RNA-seq data together by modeling the background level and saturation level of microarray probes using a nonlinear model. We modeled the microarray signal *M* as a function of the RNA-seq signal *R* with a background level *b,* a maximum saturation level *s,* and a parameter *K* indicating the RNA-seq signal at which the microarray signal is at its midpoint *(s + b) / 2*:

$$M = b + \frac{sR}{K + R} + noise.$$

We used microarray and RNA-seq data from 8 technical replicates (CT22, CT28, CT34, CT40, CT46 CT52, CT58, CT64) across all 12 tissues to fit our model. We fit the function using weighted nonlinear regression, with weights estimated from a LOESS fit on the variance and mean microarray signal for each gene across 12 time points. We constrained *b* and *s* such that adjusted values were not too close to the background or saturation levels, which would become sensitive to noise; $b_{max} = 0.8 \min(E_g)$, $s_{min} = 1.2 \max(E_g)$.

Nuclear Proteomics

Processed nuclear proteomics data of WT liver along the circadian cycle was downloaded from Supplemental Table S1 of Wang et al. 2016.

ENCODE DNase I Hypersensitivity Assays

Raw Processing

FASTQ files of liver and kidney DNAse-seq was downloaded from the mouse encode project (https://www.encodeproject.org) (Yue et al. 2014) and mapped to the mouse genome (mm9) and the signal was binned in 500 bp windows across the genome. Each isogenic replicate was assessed for quality by distribution of the log signal. We kept only samples that showed clear bimodal distribution in the binned log signal and merged replicates together for downstream analysis (Supplemental Table 5b).

Post Processing

The binned log DHS signal was converted to a z-score. A peak was specific to the liver if the zscore_liver > 3 and zscore_kidney < 0.

ChIP-exo data

FASTQ files from ChIP-exo data targeting FOXA2, ONECUT1, and REV-ERBα were downloaded from data repositories GSE57559 (GEO), PRJEB4933 (ENA), GSE67973 (GEO), respectively and mapped to mouse genome (mm9). Normalized bigwig files were visualized on UCSC track.

Data Analysis

Model selection (MS) method

We enumerated possible multiple harmonic regression models to identify rhythms in multiple tissues or genotypes and penalized models by model complexity. For each condition, the log mRNA abundance was fit with either a flat model or a rhythmic model:

$$Y_{g,c}(t) = \mu_{g,c} + \epsilon$$
$$Y_{g,c}(t) = \mu_{g,c} + \beta_{1_{g,c}} \cos(\omega t) + \beta_{2_{g,c}} \sin(\omega t) + \epsilon$$

Importantly, we allowed, for each model, $Y = X_\gamma \overrightarrow{\beta_\gamma} + \epsilon$, the possibility for rhythmic parameters in different conditions to be shared. We used a g-prior for the rhythmic parameters $\vec{\beta}$ (Liang et al. 2008),

$$\beta \sim N(0, g\sigma^2(X^T X^{-1})$$

which allows the marginal likelihood of the gene expression *E* given the model *M* to be expressed in closed form and the Bayes factor *K* can be expressed as:

$$K(M_\gamma)|g = \frac{(1+g)^{\frac{n-1-p_\gamma}{2}}}{\left(1+g(1-R^2)\right)^{\frac{n-1}{2}}}$$

Where R^2 is the coefficient of determination. We used a biased estimator of the variance $\sigma^2 = \frac{RSS}{n}$ where RSS is the residual sum of squares. The parameter g, which controls the spread of the prior over the models, was set to g=1000.

The liver and kidney RNA-seq dataset consisted of 4 conditions (WT liver, *Bmal1* KO liver, WT kidney, *Bmal1* KO kidney), which allow 52 possible rhythmic combinations. For every gene, we assigned a probability for each of the 52 models. The number of rhythmic combinations k scales as a function of the number of conditions n as $k(n) = B_{n+1}$ where B is the Bell number used in combinatorial mathematics.

In the 11 tissues dataset, we reduced the initial number of possible models from 4213597 to 700075 by restricting the models to have up to 3 independent rhythmic parameters, which can be shared amongst the 11 tissues. We used g=1000 and used an amplitude cutoff of 0.15 to assign low amplitude rhythms as nonrhythmic. Assigning each gene to one of 700075 models, 3919 models contained at least one gene.

To label tissue-wide rhythmic transcripts as clock or system-driven (Figure 2C), we assigned genes that were rhythmic in *Bmal1* KO as system-driven and genes that were rhythmic in WT only as clock-driven. One exception were genes that oscillated in *Bmal1* KO but with low amplitudes compared to WT, which we assigned as clock-driven (e.g. *Npas2*).

<u>Complex singular value decomposition (SVD) representation of gene and tissue module</u>
Gene expression over time and across tissues can be represented as a 3-dimensional array. However, since SVD of a tensor does not have all the properties of a matrix SVD, we first transformed the time domain to the frequency domain corresponding to 24-hour rhythms for all genes g and conditions c:

$$\hat{E}_{g,c} = \sum_{t \in \vec{t}} E_{g,c,t} e^{i\omega t}$$

where $\hat{E}_{g,c}$ is a complex value representing the amplitude and phase of expression for gene g in condition c and $\omega = 2\pi/24$.
The resulting matrix was decomposed using SVD and the first left -and right-singular values were visualized in separate polar plots. To ensure the first component recovered most of the original signal, the SVD representation was performed separately for each gene module identified by model selection.

Predicting Activities of Transcriptional Regulators

<u>Predictions of transcription factor binding site (TFBS)</u>
For TFBS predictions near promoters, we used motevo version 1.03 (Arnold et al. 2012) to scan +/- 500 bp around the promoter. We used promoters (Balwierz et al. 2009) and weight matrices of transcription factors defined by SwissRegulon (Pachkov et al. 2013) (http://swissregulon.unibas.ch/fcgi/sr/downloads).For distal regions, we scanned the

genome for TFBSs in 500 bp windows in genomic regions within 40 kb of an annotated gene.

Penalized regression model

We applied a penalized regression model as previously described (Balwierz et al. 2014) using an L2 penalty for penalization, which allows a direct estimate of the standard deviation. Rhythmic activities of transcription factor motifs were summarized using complex-valued singular value decomposition. We projected the activities to an amplitude and phase and calculated the zscore of the amplitude. We considered activities with zscore > 1.25 as rhythmic TF activities. Time of peak temporal activities of transcription factors were subtracted by 3 hours, to account for an average 3 hour shift between peak transcription and peak mRNA accumulation (Le Martelot et al. 2012).

Enrichment of pairs of motifs

We applied log-linear models to test for statistical significance between pairs of motifs across rhythmic versus nonrhythmic modules. For each motif, we ordered DHS sites by the posterior sitecount of the motif (decreasing order) and considered the motif to be present in the DHS site if the sitecount was in the top 300 (Myšičková and Vingron 2012). We considered liver-specific DHS sites that were annotated to a clock-dependent liver-rhythmic gene or to a nonrhythmic gene. For each annotated label and for each pair of motifs, we constructed a 2 by 2 contingency table by counting the number of DHS sites that contain one of the motifs, both motifs, or none, resulting in a 3-way contingency table (motif 1, motif 2, and annotated label). We assessed whether the resulting contingency table was statistically significant to a null model, where the null model was the expected counts if the pair of motifs were jointly independent on the rhythmicity.

Functional Analysis by GO terms

We used Fisher's exact test to assess statistical significance of gene enrichment for each GO term. Foreground genes were genes with phases within 6 hours window (e.g., phases between ZT0 to ZT6). Background genes were all genes assigned to a model (above). For each GO term, we slid the 6-hour window with a step size of 1 hour and calculated the p-value at every hour of the day. GO terms were chosen by visualizing statistically significant GO terms in the directed acyclic graph and choosing GO terms that were comparably deep in the tree.

Alternative promoter analysis

Abundances of transcripts (quantified by kallisto) with overlapping 5'UTRs were merged together as a single transcript, loosely defining them as having a "common promoter". For each gene, we compared alternative promoter usage between rhythmic tissues and nonrhythmic tissues by calculating the Euclidean distance in n-dimensional space defined by the number of transcripts n with nonoverlapping 5'UTRs.

References to Supplemental Methods

Arnold, Phil, Ionas Erb, Mikhail Pachkov, Nacho Molina, and Erik van Nimwegen. 2012. "MotEvo: Integrated Bayesian Probabilistic Methods for Inferring Regulatory Sites and Motifs on Multiple Alignments of DNA Sequences." *Bioinformatics (Oxford, England)* 28 (4): 487–94. doi:10.1093/bioinformatics/btr695.

Atger, Florian, Cédric Gobet, Julien Marquis, Eva Martin, Jingkui Wang, Benjamin Weger, Grégory Lefebvre, Patrick Descombes, Felix Naef, and Frédéric Gachon. 2015. "Circadian and Feeding Rhythms Differentially Affect Rhythmic mRNA Transcription and Translation in Mouse Liver." *Proceedings of the National Academy of Sciences* 112 (47). National Acad Sciences: E6579–88. doi:10.1073/pnas.1515308112.

Balwierz, Piotr J, Piero Carninci, Carsten O Daub, Jun Kawai, Yoshihide Hayashizaki, Werner Van Belle, Christian Beisel, et al. 2009. "Methods for Analyzing Deep Sequencing Expression Data: Constructing the Human and Mouse Promoterome with deepCAGE Data." *Genome Biology* 10 (7). BioMed Central: R79. doi:10.1186/gb-2009-10-7-r79.

Balwierz, Piotr J, Mikhail Pachkov, Phil Arnold, Andreas J Gruber, Mihaela Zavolan, and Erik van Nimwegen. 2014. "ISMARA: Automated Modeling of Genomic Signals as a Democracy of Regulatory Motifs." *Genome Research* 24 (5): 869–84. doi:10.1101/gr.169508.113.

Bray, Nicolas, Harold Pimentel, Páll Melsted, and Lior Pachter. 2015. "Near-Optimal RNA-Seq Quantification." Quantitative Methods; Computational Engineering, Finance, and Science; Data Structures and Algorithms; Genomics, May. http://arxiv.org/abs/1505.02710.

Carvalho, Benilton S, and Rafael A Irizarry. 2010. "A Framework for Oligonucleotide Microarray Preprocessing." *Bioinformatics (Oxford, England)* 26 (19). Oxford University Press: 2363–67. doi:10.1093/bioinformatics/btq431.

Dobin, Alexander, Carrie A Davis, Felix Schlesinger, Jorg Drenkow, Chris Zaleski, Sonali Jha, Philippe Batut, Mark Chaisson, and Thomas R Gingeras. 2013. "STAR: Ultrafast Universal RNA-Seq Aligner." *Bioinformatics (Oxford, England)* 29 (1). Oxford University Press: 15–21. doi:10.1093/bioinformatics/bts635.

Gheldof, Nele, Marion Leleu, Daan Noordermeer, Jacques Rougemont, and Alexandre Reymond. 2012. "Detecting Long-Range Chromatin Interactions Using the Chromosome Conformation Capture Sequencing (4C-Seq) Method." In , 211–25. doi:10.1007/978-1-61779-292-2_13.

Hagège, Hélène, Petra Klous, Caroline Braem, Erik Splinter, Job Dekker, Guy Cathala, Wouter de Laat, and Thierry Forné. 2007. "Quantitative Analysis of Chromosome Conformation Capture Assays (3C-qPCR)." *Nature Protocols* 2 (7). Nature Publishing Group: 1722–33. doi:10.1038/nprot.2007.243.

Jouffe, C, G Cretenet, L Symul, E Martin, F Atger, F Naef, and F Gachon. 2013. "The Circadian Clock Coordinates Ribosome Biogenesis." *PLoS Biol* 11 (1): e1001455. doi:10.1371/journal.pbio.1001455.

Le Martelot, Gwendal, Donatella Canella, Laura Symul, Eugenia Migliavacca, Federica Gilardi, Robin Liechti, Olivier Martin, et al. 2012. "Genome-Wide RNA Polymerase II Profiles and RNA Accumulation Reveal Kinetics of Transcription

and Associated Epigenetic Changes During Diurnal Cycles." Edited by Achim Kramer. *PLoS Biology* 10 (11). Public Library of Science: e1001442. doi:10.1371/journal.pbio.1001442.

Liang, Feng, Rui Paulo, German Molina, Merlise A Clyde, and Jim O Berger. 2008. "Mixtures of *G* Priors for Bayesian Variable Selection." *Journal of the American Statistical Association* 103 (481): 410–23. doi:10.1198/016214507000001337.

Love, Michael I, Wolfgang Huber, and Simon Anders. 2014. "Moderated Estimation of Fold Change and Dispersion for RNA-Seq Data with DESeq2." *Genome Biology* 15 (12). BioMed Central: 550. doi:10.1186/PREACCEPT-8897612761307401.

Myšičková, Alena, and Martin Vingron. 2012. "Detection of Interacting Transcription Factors in Human Tissues Using Predicted DNA Binding Affinity." *BMC Genomics* 13 (Suppl 1). BioMed Central: S2. doi:10.1186/1471-2164-13-S1-S2.

Pachkov, Mikhail, Piotr J Balwierz, Phil Arnold, Evgeniy Ozonov, and Erik van Nimwegen. 2013. "SwissRegulon, a Database of Genome-Wide Annotations of Regulatory Sites: Recent Updates." *Nucleic Acids Research* 41 (Database issue): D214-20. doi:10.1093/nar/gks1145.

Ripperger, Jürgen A, and Ueli Schibler. 2006. "Rhythmic CLOCK-BMAL1 Binding to Multiple E-Box Motifs Drives Circadian Dbp Transcription and Chromatin Transitions." *Nature Genetics* 38 (3). Nature Publishing Group: 369–74. doi:10.1038/ng1738.

Wang, Jingkui, Daniel Mauvoisin, Eva Martin, Florian Atger, Antonio Núñes Galindo, Loïc Dayon, Federico Sizzano, et al. 2016. "Nuclear Proteomics Uncovers Diurnal Regulatory Landscapes in Mouse Liver." *Cell Metabolism*. doi:10.1016/j.cmet.2016.10.003.

Yue, Feng, Yong Cheng, Alessandra Breschi, Jeff Vierstra, Weisheng Wu, Tyrone Ryba, Richard Sandstrom, et al. 2014. "A Comparative Encyclopedia of DNA Elements in the Mouse Genome." *Nature* 515 (7527). Nature Publishing Group, a division of Macmillan Publishers Limited. All Rights Reserved.: 355–64. doi:10.1038/nature13992.

Zhang, Ray, Nicholas F Lahens, Heather I Ballance, Michael E Hughes, and John B Hogenesch. 2014. "A Circadian Gene Expression Atlas in Mammals: Implications for Biology and Medicine." *Proceedings of the National Academy of Sciences of the United States of America* 111 (45): 16219–24. doi:10.1073/pnas.1408886111.
